# Method Matters: Exploring Alkoxysulfonate-Functionalized Poly(3,4-ethylenedioxythiophene) and Its Unintentional Self-Aggregating Copolymer toward Injectable Bioelectronics

**DOI:** 10.1021/acs.chemmater.1c04342

**Published:** 2022-02-28

**Authors:** Abdelrazek H. Mousa, David Bliman, Lazaro Hiram Betancourt, Karin Hellman, Peter Ekström, Marios Savvakis, Xenofon Strakosas, György Marko-Varga, Magnus Berggren, Martin Hjort, Fredrik Ek, Roger Olsson

**Affiliations:** † Department of Chemistry and Molecular Biology, University of Gothenburg, 405 30 Gothenburg, Sweden; ‡ Chemical Biology & Therapeutics, Department of Experimental Medical Science, Lund University, 221 84 Lund, Sweden; § Division of Oncology, Department of Clinical Sciences, Lund University, 221 84 Lund, Sweden; ∥ Laboratory of Organic Electronics, Department of Science and Technology, Linköping University, 601 74 Norrköping, Sweden; ⊥ Division of Clinical Protein Science & Imaging, Department of Clinical Sciences and Department of Biomedical Engineering, Lund University, 221 00 Lund, Sweden; # Department of Translational Medicine, Lund University, Skåne University Hospital Malmö, 202 13 Malmö, Sweden

## Abstract

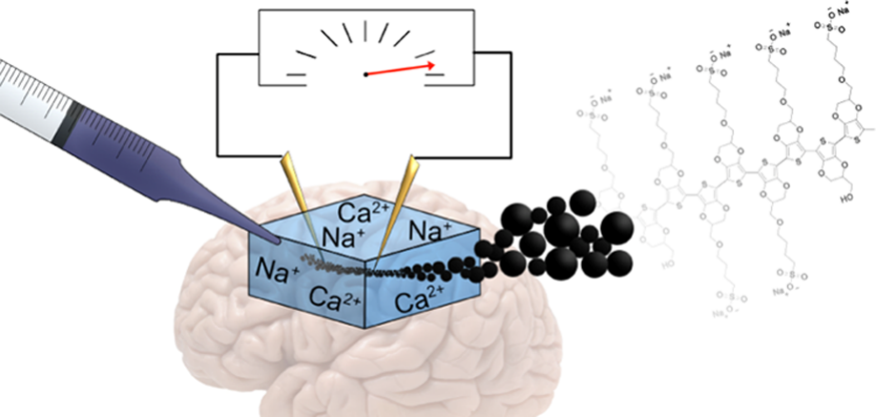

Injectable bioelectronics could become an alternative or a complement to traditional drug treatments. To this end, a new self-doped p-type conducting PEDOT-S copolymer (**A5**) was synthesized. This copolymer formed highly water-dispersed nanoparticles and aggregated into a mixed ion–electron conducting hydrogel when injected into a tissue model. First, we synthetically repeated most of the published methods for PEDOT-S at the lab scale. Surprisingly, analysis using high-resolution matrix-assisted laser desorption ionization-mass spectroscopy showed that almost all the methods generated PEDOT-S derivatives with the same polymer lengths (i.e., oligomers, seven to eight monomers in average); thus, the polymer length cannot account for the differences in the conductivities reported earlier. The main difference, however, was that some methods generated an unintentional copolymer P(EDOT-S/EDOT-OH) that is more prone to aggregate and display higher conductivities in general than the PEDOT-S homopolymer. Based on this, we synthesized the PEDOT-S derivative **A5**, that displayed the highest film conductivity (33 S cm^–1^) among all PEDOT-S derivatives synthesized. Injecting **A5** nanoparticles into the agarose gel cast with a physiological buffer generated a stable and highly conductive hydrogel (1–5 S cm^–1^), where no conductive structures were seen in agarose with the other PEDOT-S derivatives. Furthermore, the ion-treated **A5** hydrogel remained stable and maintained initial conductivities for 7 months (the longest period tested) in pure water, and **A5** mixed with Fe_3_O_4_ nanoparticles generated a magnetoconductive relay device in water. Thus, we have successfully synthesized a water-processable, syringe-injectable, and self-doped PEDOT-S polymer capable of forming a conductive hydrogel in tissue mimics, thereby paving a way for future applications within in vivo electronics.

## Introduction

Bioelectronics, which aims to modulate and monitor biological processes, is an evolving field complementing traditional small-molecule drug treatments and other new modality strategies.^1^ Moving beyond the current stimulation techniques used, for example, in Parkinson’s disease, epilepsy, vagus nerve stimulation, and pain,^2−5^ there is a need to develop minimally invasive implantable bioelectronic devices without foreign body responses. A recent study suggested that, in order to avoid blood vessel rupture and bleeding in the brain, a syringe needle with diameter below 25 μm should be used for implantation of bioelectronics.^6^ To meet this goal, we envisioned a strategy that uses highly soluble nanoparticles that self-organize into a conducting structure when injected into a tissue, by using a capillary with a diameter of about 25 μm. Injectable conductive hydrogels have shown great biocompatibility because of their resemblance to body tissue. Conductive hydrogels are in general composed of a hydrogel matrix (e.g., alginate, hyaluronic acid, or chitosan) supplemented by a conductive polymer (e.g., PEDOT:PSS, polypyrrole, or oligo-/polyaniline) and have conductivities in a low mS cm^–1^ range.^7,8^ In the search for a material that allows for the installation of different functional groups to diversify functionality toward controlling solubility–aggregation properties and biocompatibility, the polymer poly(3,4-ethylene-dioxythiophene) doped with poly(styrene sulfonate) (PEDOT:PSS)^9−11^ was of interest. PEDOT:PSS displays high conductivity and the possibility for structural manipulation of the EDOT monomer.^12−16^ Recently, a mixture of PEDOT:PSS and 4-dodecylbenzenesulfonic acid (DBSA) was reported to form hydrogels at room temperature after 2–200 min, depending on the concentration of DBSA. This hydrogel formation was demonstrated by extruding a hydrogel with a syringe on a >400 μm diameter scale, and it showed a conductivity of about 10^–1^ S cm^–1^, which is at the higher end of hydrogel conductivities^17^ and higher than most tissues (<10^–2^ S cm^–1^).^18^ However, the hydrophilic two-component pristine PEDOT:PSS can disintegrate and lose function when exposed to excessive humidity or water because of the highly water-soluble PSS component.^11^ To reduce the number of crucial components for function and the chemical bulk, we sought to remove the nonconductive bystander materials in the injectable solution, that is, PSS and DBSA. An interesting solution to this is the self-doped polythiophenes, for example, PEDOT derivatives with a covalently attached sulfonate group known as PEDOT-S. PEDOT-S derivatives have been reported as having high conductivities from a few mS cm^–1^ up to 1089 S cm^–1^, a high aqueous solubility of >40 mg/mL, and high biocompatibility.^19−21^ In addition, PEDOT-S has been synthesized by electropolymerization^22,23^ and oxidative polymerization; in a normal organic lab setting, the latter is an attractive option because of its simplicity, low cost, and scalable synthesis.

As an emerging alternative conductive polymer to PEDOT:PSS, there have been several reported methods for the oxidative polymerization of EDOT-S^19,21,23^ and closely related S-EDOT monomers^20^ into PEDOT-S and S-PEDOT, respectively. These methods typically use Fe(III) or Fe(II), either as a stoichiometric oxidant or in catalytic amounts in the presence of a persulfate as a stoichiometric oxidant. Different solvents, reactants, reaction times, temperatures, and workups have been reported. More recently, examples of direct C–H arylation polymerization reactions targeting the synthesis of controlled copolymers of PEDOT analogues, as well as the PEDOT-S homopolymer, have been reported.^24−26^

Although there are few publications on PEDOT-S compared with those focusing on PEDOT:PSS, deciphering the properties (e.g., polymer size, solubility, and conductivity) of the synthesized materials using different methods—including postprocessing, which is aimed at specific utilities—just by reading the literature is a challenge. Therefore, we decided to reproduce six original protocols that we found in the literature at a lab scale (we are now aware of a couple of more protocols, but those procedures are mostly covered within the different procedures in the current work) and compare them in a setting applicable for our use: highly soluble self-organizing nanoparticle dispersions based on PEDOT-S forming a conductive hydrogel in a biologically relevant environment. We describe the discovery that some methods generated PEDOT-S/-OH heteropolymers not PEDOT-S homopolymers. The importance of this heterogenicity for solubility and self-aggregation was evaluated and used to generate an injectable hydrogel displaying high conductivities (1–5 S cm^–1^) in a model system.

## Experimental Section

Details about the synthesis and characterization of previously reported monomers, polymerization methods, variations of the **A5** method, and synthesis of EDOT-TMA and PEDOT-TMA can be found in the Supporting Information.

### Synthesis of PEDOT-S Using Okuzaki’s Method^20^ (**A5**)

EDOT-S (300 mg, 0.908 mmol) and Fe(II)SO_4_·7H_2_O (0.6 equiv, 151 mg, 0.543 mmol) were added to a reaction vial, that was capped and flushed with nitrogen. 1 M aq. H_2_SO_4_ (5 mL) was added, and nitrogen flushing continued during addition. (NH_4_)_2_S_2_O_8_ (2 equiv, 416 mg, 1.822 mmol) was dissolved in 1 mL of Milli-Q water, giving a clear solution that was added dropwise using a syringe to the above solution, causing a color change: first to green and then to deep blue. The reaction mixture was stirred at rt for 20 h under nitrogen. The mixture turned viscous within 2 h after the addition of ammonium persulfate. After 20 h, the crude material was diluted with 10 mL of water. Cation exchange resin (16 g, Lewatit Monoplus S108H) was rinsed by shaking in Milli-Q water (2 × 20 mL). The resin was collected by filtration and added to the reaction mixture, which was shaken overnight (shaking table). The cation resin was then removed by filtration and washed with water (3 × 10 mL); the collected deep blue aq. solution was poured into a glass vial containing anion exchange resin (16 g, Lewatit mp62, free base) previously rinsed as for the cation exchange resin. The mixture was shaken overnight. The anion exchange resin was then removed by filtration, washed with Milli-Q water (3 × 10 mL), and the resulting deep blue solution was freeze-dried, giving 214 mg (71% yield) of the polymer as a dark blue solid.

### Preparation of CY3-Tagged Polyornithine

Cyanine3 NHS ester (Lumiprobe, 1 mg in 50 μL DMSO) was added to polyornithine hydrobromide (Sigma-Aldrich P3655, 10 mg) in 1 mL Ringer medium (pH 7.25). After 1 h, the reaction mixture was spin-filtered (0.22 μm), and the final solution was used without further purification.

### Characterization of PEDOT-S Diffusion

PEDOT-S (10 mg/mL, 4 μL) and polyornithine-CY3 (10 mg/mL, 4 μL) were injected using a 10 μL Hamilton syringe into an agarose gel (0.5% in Ringer buffer or Milli-Q water) in 6-well microtiter plates with a 15 mm separation (spot size 2 mm). After 2 h, the size of the spot was measured again. After 15 days, the diffusion ratio was measured as the distance between the PEDOT-S injection spot and the polyornithine-PEDOT precipitation divided by the total distance between the PEDOT-S and polyornithine-CY3 injections.

A diffusion ratio for PEDOT-S and polyornithine (CY3) of 0.5 would mean an equal diffusion ratio.

### Formation of an **A5** Wire

The **A5** polymer solution (50 μL, 10 mg/mL in Milli-Q water) was added into isopropanol (20 mL) in a Petri dish, which resulted in a polymer film at the bottom surface. After 10 min, the film was lifted using a tweezer upon which it spontaneously folded into a wire. The wire was then dried for 1 h in air.

### Cross-Linking of the Wire

The **A5** wire from the previous step was dipped into a solution with 0.1 M CaCl_2_. The cross-linked **A5** wire was then air-dried for 2 h before analysis.

### Formation of a Magnetic **A5** Wire with Fe_3_O_4_ Nanoparticles

Using the methods above (formation of the **A5** wire, followed by cross-linking of the wire), 75 μL (10 mg/mL) **A5** and 25 μL (5 mg/mL) iron nanoparticles (Fe_3_O_4_, 5 nm, Sigma-Aldrich), were mixed to form an **A5**-wire containing the magnetic nanoparticles. The cross-linked wire was confirmed to be magnetic using a neodymium magnet.

### High-Resolution MALDI-MS Measurements

Matrix-assisted laser desorption ionization (MALDI) mass spectra (MS) were obtained on an LTQ Orbitrap XL (Thermo Scientific, Bremen, Germany). PEDOT-S polymers were dissolved in 0.1% TFA at a concentration of 0.2 mg/mL. The MALDI matrix 2, 5-dihydroxybenzoic (DHB) acid was prepared at 10 mg/mL in 50:50 [v/v] methanol/0.1% TFA in water. The PEDOT-S solution (0.5 μL) was air-dried on the flat surface of a stainless steel plate. Next, 0.5 μL of DHB was deposited over the PEDOT layer, and the mixture was allowed to dry. Full mass scans were acquired in positive mode in the *m*/*z* range of 800–4000 Th at 15,000 resolution using a FT mass analyzer (Orbitrap). Twenty laser shots were employed for each analysis with a laser energy set to 30 μJ. The MS were processed using Xcalibur software v3.0.63 (Thermo Scientific).

### Gel Permeation Chromatography

Gel permeation chromatography (GPC) was performed by PSS Polymer Standards Service GmbH. Details of running conditions, chromatograms, and molecular weight distributions are given in the Supporting Information.

### Electrical Measurements

A Keithley 2612B connected to Au-coated W electrodes (Cat: SE-TG, Signatone, Gilroy, CA) was used to characterize the electrical properties. For the dried samples, a 3 μL droplet [10 mg/mL] of the PEDOT-S dissolved in Milli-Q water was placed on a glass slide. After drying in air, the PEDOT-S was contacted at an electrode distance of 2.5 mm. An applied bias was swept between −0.5 and +0.5 V and the current was measured. Linear current–voltage relationships were typically recorded. The conductivities of **A5** wire in water and the PEDOT-S in agarose were measured in a similar manner; however, in this case, current–voltage sweeps were performed at several electrode distances. The transmission line model was used to extract the conductivity of the polymers.

### Four-Point Probe and Four Patterned Au Line Measurements

The polymers were diluted in deionized water at a final concentration of 10 mg/mL, and the solution was sonicated for 1 h. The sonicated solution was spin-coated on top of either parylene-C coated glass slides or on patterned Au lines with a width of 2.5 mm, a length of 15 μm, and a distance between each electrode of 15 μm. The fabrication of the electrodes was based on a previously reported parylene fabrication process.^27^ The area of the **A5** film was defined by peeling a sacrificial layer of parylene C by using a peel-off technique. For the four-point probe measurements, the conductivity was calculated from the sheet resistance
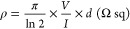
1where *I* is the applied current at the outer probes and *V* is the measured voltage at the inner probes. *d* is the thickness of the films, and it was measured using a Dektak profilometer. The conductivity was then *g* = 1/ρ (S cm^–1^). Similarly, the conductivity caused by using patterned Au lines was calculated by the geometrical characteristics of the films
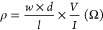
2where *w* is the width and *l* is the length. The conductivity was calculated using *g* = 1/ρ (S cm^–1^).

For organic electrochemical transistor (OECT) measurements, phosphate buffered saline (PBS) was drop-casted on top of the **A5** films, and an Ag/AgCl pellet was immersed in the PBS electrolyte.

## Results and Discussion

**Scheme 1:**
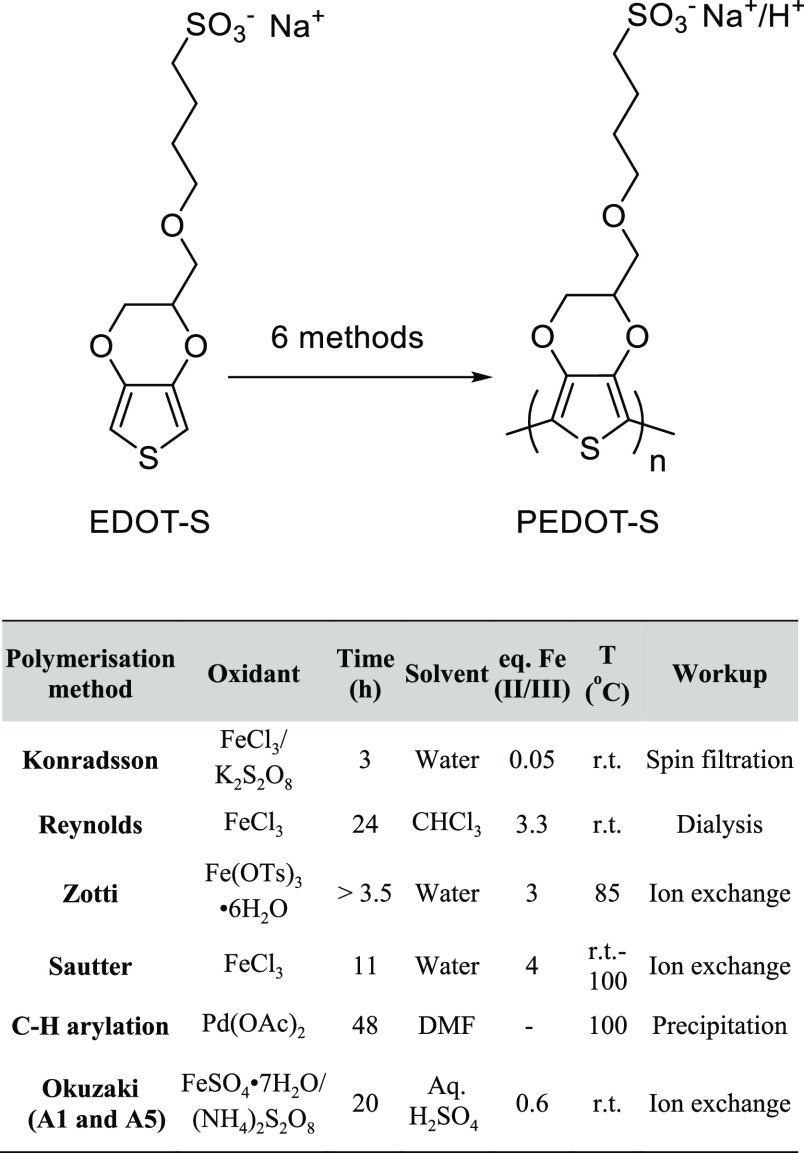
Summary of Methods Used in This Study for PEDOT-S Synthesis

With the aim of finding a PEDOT-S formulation suitable for biomedical applications, we reproduced six previously reported synthetic methods for PEDOT-S by chemical oxidative polymerization of EDOT-S (Scheme 1).

To facilitate the discussion, the methods are represented by the corresponding author (Zotti,^23^ Reynolds,^21^ Konradsson,^19^ and Okuzaki^20^) and an inventor in the case of a patent method (Sautter^28^). For clarity, the only iron-free method is denoted as C–H arylation (Yu^26^). Furthermore, the original Okuzaki method produced PEDOT with butane-2-sulfonate, which is denoted S-PEDOT. For comparison with the other methods, we have used the Okuzaki method to synthesize PEDOT-S with butane-1-sulfonate, which we named **A5** and **A1** (5 and 1 stand for the percentage of the concentration of the monomer in the reaction mixture) to see if there was a correlation in the method using the butane-1-sulfonate compared with butane-2-sulfonate, the one used by Okuzaki. In addition, the original S-PEDOT synthesis was also reproduced.

Five of the methods use Fe(III) as an oxidant, added to the reaction mixture as Fe(III) or Fe(II), either in a catalytic amount (Konradsson), in a 3–4 equiv excess (Sautter, Zotti and Reynolds) or 0.6 equiv (Okuzaki). In addition, the Konradsson and Okuzaki methods use persulfate as a stoichiometric oxidant. Except for the Reynolds method, which is performed in chloroform, all methods use water as the solvent (Scheme 1). Different workup procedures are used to remove excess salt and monomers from the material after synthesis. For example, the Sautter, Zotti, and Okuzaki methods all use treatment with ion exchange resins (cationic and anionic resins) to purify the polymer, and the Reynolds method uses dialysis. As an alternative to iron-catalyzed oxidative polymerization, a palladium-catalyzed polymerization of EDOT-S and its dibromo derivative, which have been reported to give high-molecular-weight polymers,^25,29^ was also included in our study. The synthetic parameters and workup methods are summarized in Scheme 1.

**Figure 1:**
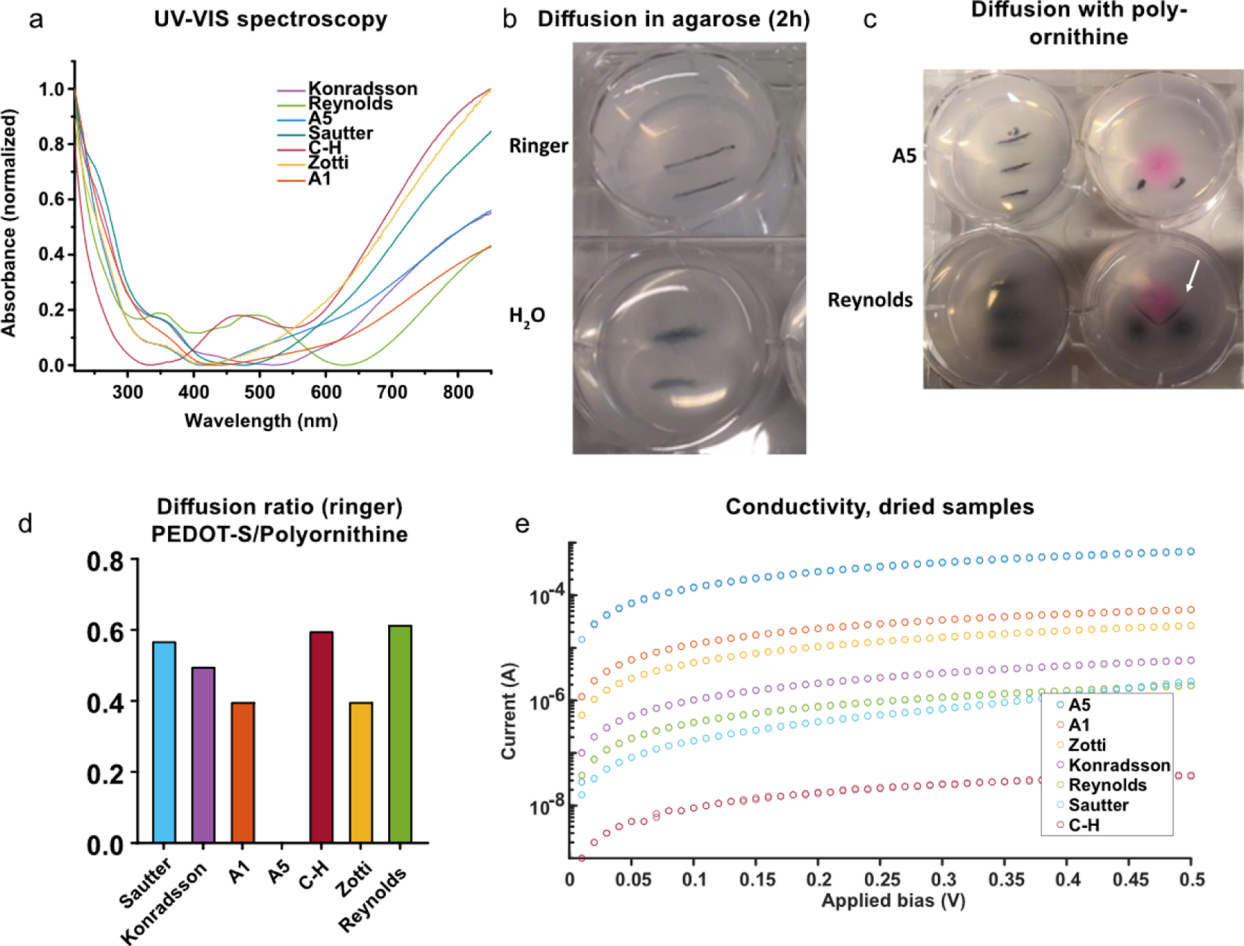
PEDOT-S synthesis evaluation. (a) UV–vis absorption spectra of the PEDOT-S polymer prepared using different polymerization methods. (b) Photograph depicting the lack of **A5** diffusion in Ringer-agarose and substantial diffusion in water agarose. (c) Photograph of the representative diffusion experiments in agarose 0.5% (Ringer solution pH 7.2); left: injection of PEDOT-S (10 mg/mL, 4 μL), right: injection of PEDOT-S (10 mg/mL, 4 μL) and polyornithine tagged with CY3 (10 mg/mL, 4 μL). Please note the formation of black lines between the PEDOT-S and PO for the Reynolds variant, as indicated by the white arrow. (d) Diffusion rate between the PEDOT-S and tagged polyornithine when injected into Ringer-agarose. **A5** shows limited diffusion. (e) Two-terminal electrical conductivity measurements mapped from 3 mm dried samples of the PEDOT-S variants.

The five iron-catalyzed EDOT-S polymerization methods (see the Supporting Information for synthetic details) yielded materials that resulted in dark blue solutions when dissolved in deionized water. The palladium catalyzed method resulted in a material that gave a brown-colored solution. This could be because of the workup method used to purify the polymer, which only uses precipitation with acetone, which, in our experience, is not enough to remove the excess salt used in this method. The initial characterization of the reproduced PEDOT-S materials included UV–vis absorption spectroscopy (Figure 1a).

The absorption UV–vis spectra for all the PEDOT-S materials displayed broad absorption peaks in the near-IR region (>700 nm), which is typically observed for doped PEDOT polymers.^30^ For the Reynolds method, which uses 1 M NaOH in the workup, a broad peak at 500 nm corresponding to the dedoped polymer was observed. The dedoping effect of NaOH was reported by Reynolds in the original publication,^21^ and the effect significantly decreased after dialysis. Dialysis of the NaOH-treated PEDOT-S resulted in a color change from purple to the blue color observed for the doped PEDOT-S.

For the polymer to be injectable and form a structure that is conductive in vivo, the balance between solubility and diffusion in vivo is key. To increase the likelihood of self-aggregation into a conductive structure, the nanoparticles need to have high aqueous solubility while demonstrating limited diffusion, but still enough diffusion to be able to occupy the backtrack (column injection) of the capillary trace and to reach nearby cells for seamless infiltration.

As an initial evaluation of the diffusion properties, we studied the diffusion of all the prepared PEDOT-S materials after injection into an agarose gel^31^ (0.5%, a surrogate for brain tissue) prepared with Milli-Q water or Ringer buffer, the latter is a water solution of physiological salt concentrations mimicking body fluids. Aqueous solutions of PEDOT-S were injected in a 0.5 wt % agarose gel cast in Milli-Q water. All PEDOT-S derivatives showed complete diffusion; after 2 h, the distinct pattern from the injection site had transformed into a gray cloud in the gel (Figure 1b). Because PEDOT-S is an anionic polyelectrolyte, it can be expected to precipitate in the presence of a polymeric amine base such as polyornithine. Injection was performed in an agarose gel cast in Ringer buffer at a set distance from the injection of a fluorescently tagged polyornithine (Figure 1c). Both polymers will diffuse through the agarose gel, forming a black line at the point of interception. The position of the black line in relation to the injection points will give information about the diffusion behavior of the material. Here, **A5** showed limited diffusion, whereas the PEDOT-S materials from all the other methods diffused to various degrees (Figure 1d).

Having established the formation of stable distinct thread-like structures within the agarose gel with **A5**, the next step was to determine the conductive properties of the PEDOT-S materials. The goal is to use these polymers in vivo, therefore we set out to evaluate the conductivity of the polymers using a two-terminal setup without postprocessing. However, because of the high diffusion in agarose for most PEDOT-S derivatives, it was only possible to get a reading from **A5**. The conductivity was estimated to be in the range of 1–5 S cm^–1^, here depending on the estimation of its exact geometry in the gel. Therefore, 3 μL droplets containing the polymers [at 10 mg/mL] were left to dry on a glass cover slide. Once dried, an external voltage was swept between two Au electrodes, and the current flowing between the electrodes was registered (Figure 1e). This method provides a rough estimate of the polymer’s conductivity, but it fails to capture the subtle nuances available with more advanced methods such as four-point probe setups or impedance measurements, where contact resistances can be excluded. However, this method is well-suited for ranking the different polymers in a relevant setting.

We found that the conductivity strongly depended on the synthetic procedure, which spanned more than 4 orders of magnitude (Figure 1e). All polymers showed similar, linear current–voltage characteristics but shifted in magnitude, except for the Sautter version, which deviated slightly at voltages larger than 0.2 V. Here, **A5** had the highest conductivity. A second group consisting of **A1** and Zotti (reported conductivity of 1–5 S cm^–1^) showed intermediate performance. Second to last came a group comprising Konradsson (reported conductivity of 12 S cm^–1^) and Sautter and Reynolds (reported conductivity of 0.2 mS cm^–1^). At the very last with the lowest conductivity came the C–H arylation method. Except for the Konradsson PEDOT-S, the ranking of the PEDOT-S conductivities followed the reported conductivities, if taking the freedom to expect the same conductivity for **A5** synthesized by the Okuzaki method as for the original S-PEDOT (1089 S cm^–1^). However, our measurements did not include any post-treatments that might have been used in the original procedures. In addition, to figure out the contribution of different reagents in the Okuzaki method, different PEDOT-S were produced, iteratively removing one reactant at a time. In one case, removing the acid from the methodology produced a material (**A5Y**) with a conductivity that was similar to those of **A1** and Zotti. This method resembles a later method published by Konradsson and Berggren (reported conductivity of 30 S cm^–1^).^32^

To conclude, we found that **A5** had the highest conductivity in the dry state and was the only polymer that rendered a conductive structure when injected into agarose, thereby making it an excellent candidate for syringe-injectable bioelectronics.

Because the Okuzaki method has not previously been reported for the synthesis of PEDOT-S (**A5**), a four-point probe measurement of conductivity was carried out in a laboratory blinded to sample identity (Table 1). The conductivity of **A5** was measured after spin-coating and drying a 200 nm thick film. Measurements using two different methods (Figure S19) gave a conductivity of 33 S cm^–1^. The Zotti and Konradsson methods were also analyzed using the same setup, mainly because of the discrepancy in the rank order between our two-point measurements and published values. Here, the four-point probe measurements gave 3 S cm^–1^ for Zotti, which is between the 1 and 5 S cm^–1^ published, and for Konradsson 6 S cm^–1^, which is still two times lower than what has been published. However, these results corroborate with the rank order of the published results, indicating that the Konradsson PEDOT-S forms better films in this setting. For comparison, we also synthesized the S-PEDOT originally made by Okuzaki and co-workers.^20^ This polymer has a high reported conductivity (1089 S cm^–1^). The monomer was synthesized using the same protocol as that used for EDOT-S with 2,4-butane sultone in place of 1,4-butane sultone. The S-PEDOT showed a conductivity of 30 S cm^–1^, which is basically the same as that of **A5**.

**Table 1:** Four-Point Probe Measurements for Selected PEDOT-S Materials

material	conductivity (S cm^–1^)^a^	average length^b^	maximum length^c^	EDOT-OH/S^f^
**A5**	33	7–8	12	15/85
**A5** + Fe^3+^^d^	19	7–8	12	
**A5** wt 10% EDOT-OH	15	7–8	12	25/75
**A5** + Fe n.p.^e^	0.39	7–8	12	
Zotti	3	7–8	11	10/90
Konradsson	6	5	9	5/95
S-PEDOT (Okuzaki)	30	7–8	12	trace

a Average of three measurements.

b Average length by the number of monomer units estimated in MALDI-MS.

c Maximum length by the number of monomer units observed in MALDI-MS.

d 1 mM Fe^3+^ added to **A5** postpolymerization.

e 15 wt % of 5 nm Fe_3_O_4_ nanoparticles added to **A5** postpolymerization.

f Ratio between EDOT-OH and EDOT-S estimated from MALDI-MS.

**A5** was also tested as a channel on OECTs, with an Ag/AgCl pellet being the gate. OECTs are three terminal devices in which the channel and gate are immersed in the electrolyte. OECTs are used in biological applications because of their current transduction properties.^33^ After spin coating **A5** with a thickness of 200 nm, the area of the channels was defined using a parylene peel-off technique^34^ with a width of 100 μm and a length of 10 μm. The channels exhibited a typical OECT behavior, as shown in IV (Figure S20B), providing a stable performance in aqueous environments. The peak transconductance was found to be at *V*_g_ = −0.3 V (Figure S20C) with a value of 1.2 mS, which is relatively high for a polymer without additional dopants.^35^ Having identified a synthetic procedure for PEDOT-S that resulted in a polymer that formed conductive structures in the agarose model, we turned our attention to better understanding the reason behind the differences between the various PEDOT-S preparations. An initial hypothesis was that the polymer length was the reason for these differences. This was also brought forward by Okuzaki as an explanation of the high conductivity found when using their methodology. Thus, a size comparison of the different PEDOT-S materials was performed to search for a possible correlation between the polymer length and diffusion, conductivity, or both. Size determination of PEDOT-S is typically carried out either by GPC, MALDI-MS, or both. GPC has been reported to overestimate the size of thiophene-based polyelectrolytes similar to PEDOT-S because of an expansion of the polymer chain caused by counterion dissociation and the associated charge repulsion between the charges in the material.^36^ Furthermore, PSS is normally used as a reference for size determination in GPC. The PSS backbone incorporates sp^3^-hybridized carbons, whereas the backbone of PEDOT-S has mainly sp^2^-hybridization; thus, PSS can adopt conformers similar to a yarn ball, while PEDOT-S has a different extended structure because of the rigid π-conjugated backbone and electrostatic repulsion between the alkoxysulfonate side chains. Furthermore, strong molecular aggregations also have an impact on the GPC analysis.

In contrast, MALDI-MS is expected to underestimate the size distribution of longer polymers because of higher efficiency in producing gas-phase ions of lower molecular mass.^37^ With these limitations in mind, high-resolution MALDI-MS can still provide a comparison within a series of polymers and was used in this study to determine and compare the molecular size of PEDOT-S obtained using different methods. We found that except for the Konradsson methodology, all other iron-catalyzed methods, including the methodology variations based on the Okuzaki method, for example, **A5Y** (Supporting Information), gave essentially the same size: an average polymer length of seven to eight monomers and a maximum length of 11 (Reynolds and Zotti) and 12 (Sautter and **A5**) monomers. Konradsson’s protocol gave polymers with average of five monomers and a maximum length up to nine monomer units (see the Supporting Information for the high-resolution MALDI-MS spectra). This corroborates the MALDI-MS analysis reported by Zotti of six to eight monomer units on average and polymers of up to 15 monomer units. For the palladium-catalyzed polymerization, we were unable to detect any oligomers on MALDI-MS, indicating that the C–H method did not produce PEDOT-S; the method was repeated several times with the same outcome. Contrary to this observation, the UV–vis spectrum indicated the presence of doped oligomers (Figure 1a). This might be because of the presence of trace amounts of oligomers, dimers, and trimers in this synthesis, hence giving rise to the UV–vis signal. A similar observation was recently noted using direct (hetero) arylation polymerization, where using both steric exclusion chromatography and MALDI-ToF MS, only dimers and trimers were detected.^24^

Our intention was to compare MALDI-MS with GPC of a set of PEDOT-S (**A5**, Konradsson, and Sautter). These polymers were sent to an external contract organization for GPC analysis using both UV (254 nm) and refractive index (RI) detectors. However, a large polymer dispersion index was seen, mainly because of *M*_w_, in the order of 100,000 Da, which is an indication of aggregation. Surprisingly, scanning the literature, except for the Okuzaki S-PEDOT publication, we were unable to find any raw data (chromatograms, *M*_w_-distribution, or both) in the literature on GPC of PEDOT-S. However, the unpublished results^38^ showed a chromatogram of *M*_w_ distribution using a RI detector that gave the GPC results of *M*_n_ = 28,000 and *M*_w_ = 123,000, which resemble our data. In our analysis, the *M*_n_ values looked reasonable: **A5** (*M*_n_ 5080 Da, *M*_w_ 174,000 Da), Konradsson (*M*_n_ 6040 Da, *M*_w_ 66,000 Da), and Sautter (*M*_n_ 7200 Da, *M*_w_ 72,000 Da). The GPC *M*_n_ for **A5** is in the same ballpark as reported for Okuzaki’s S-PEDOT. A time dependence on the GPC results was also noticed, where *M*_n_, *M*_w_, or both, increased when the samples were left for 12 and 24 h, prior to analysis (see the Supporting Information). It is interesting that when using a RI detector, the *M*_n_ values were doubled compared with UV (254 nm), while the *M*_w_ values were similar; thus, the RI detector seems to overestimate low-molecular-weights (Supporting Information). In addition, Konradsson and Berggren analyzed PEDOT-S using GPC analysis (*M*_n_ = 1700 and *M*_w_ = 5500), indicating an average monomer length of five EDOT-S units, which is somewhat shorter than our analysis of **A5Y**. Dynamic light scattering measurements performed on **A5** in a 1 mM NaCl solution showed the presence of nanoparticles with a diameter of 81 ± 32 nm in size (distribution by the number, see the Supporting Information). These results further corroborate the hypothesis that **A5** forms aggregates in solution.

**Figure 2:**
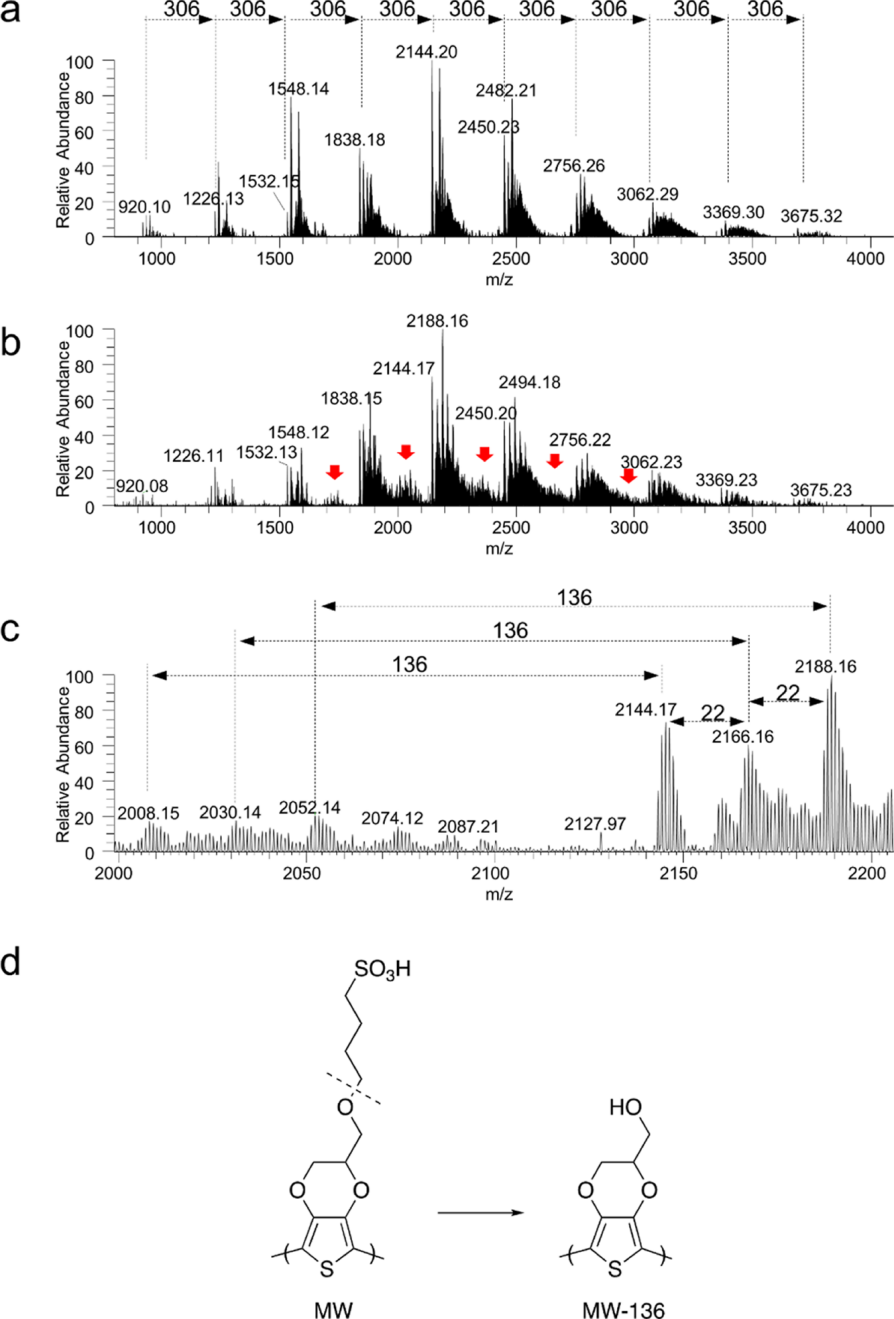
Analysis by high-resolution MALDI-MS of PEDOT-S polymers. (a) MALDI-MS of the polymer obtained using the Sautter FeCl_3_ method. (b) MALDI-MS of **A5** with red arrow marks, signaling the heterogeneities generated during the synthesis. (c) Mass range 2000–2200 Da of the **A5** spectrum showing the loss of 136 Da from the main polymer signals. The signals separating 22 Da correspond to the Na adduct. (d) Heterogeneities detected in **A5** were assigned to the loss of butanesulfonic acid (−136 Da) during the synthesis.

Because the size difference was negligible between the different methods (see the Supporting Information for the MALDI spectra), this could not explain the difference in diffusion and conductivity. However, using high-resolution MALDI-MS, heterogeneity was identified in most of the methods, except for the ones using an excess FeCl_3_ (Reynolds and Sautter), without the addition of any acid or persulfate (Figures 2a and S6). A closer analysis of the MS showed that the obtained polymers in (Konradsson, Zotti, **A5**, and **A5Y**), rather than PEDOT-S, are a PEDOT-S/OH copolymer with various degrees of EDOT-OH incorporation (Figure 2b–d). The methods using persulfate showed most heterogeneity in the polymer, and the acid had little to no additional impact as **A5Y** also showed this heterogeneity.

**Scheme 2:**
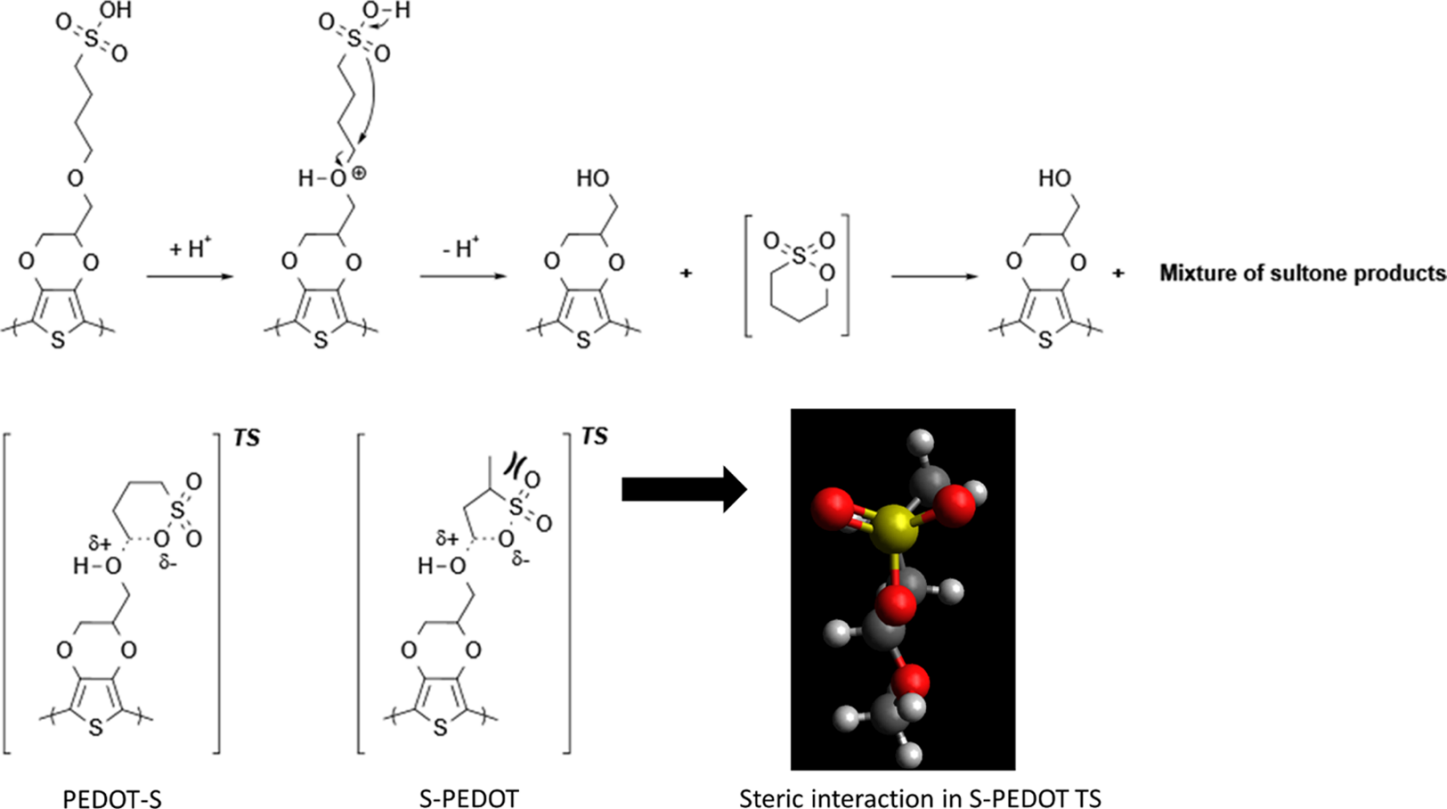
Mechanism for the Formation of EDOT-OH in PEDOT-S vs S-PEDOT

Only trace amounts of the copolymer with EDOT-OH were observed in the high-resolution MALDI-MS (Figure S9) of S-PEDOT; however, S-PEDOT had the same diffusion characteristics as **A5** in Ringer cast agarose. Interestingly, the MALDI-MS analysis showed an S-PEDOT length distribution similar to that of other PEDOT-S; thus, the methodology did not give the longer polymers as previously presented in our hands. Thus, the same conditions lead to substantial formation of EDOT-OH monomeric units in PEDOT-S, but not in S-PEDOT. One conceivable mechanism that could explain these results would be an intramolecular acid-catalyzed ether hydrolysis (Scheme 2). Whereas the EDOT-S monomeric unit forms a transition state with a 6-membered ring structure with limited steric interactions, the S-EDOT forms a 5-membered transition state with increased steric interaction between the methyl and sulfonate groups (the methyl group is almost eclipsed with the oxygen). This could explain why S-PEDOT is less prone to hydrolysis and contain less EDOT-OH monomeric units.

The methyl substituent on the linker might shield the sulfonic acid, preventing strong charge repulsions and, thus, giving a stronger aggregation effect, despite that only trace amounts of EDOT-OH are present in S-PEDOT compared with **A5**.

Interestingly, Zotti using only Fe(OTs)_3_ gave some EDOT-OH content, but less compared to using persulfates. The mechanism for this reaction needs to be further investigated.

**Figure 3:**
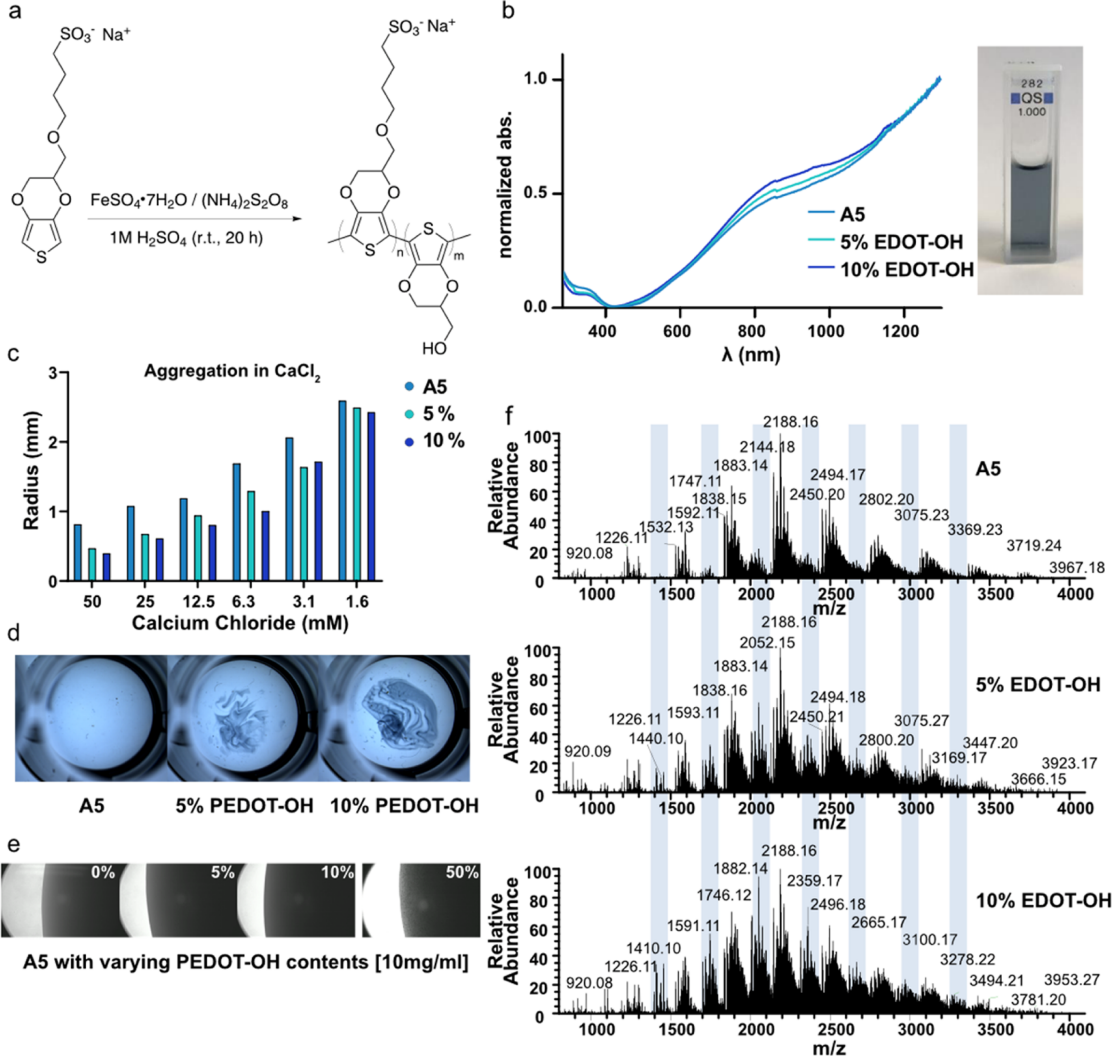
**A5** characterization (a) reaction scheme, (b) left, UV–vis spectra of **A5** and **A5** with 5 and 10% EDOT-OH added in the polymerization, and right the photograph of **A5** dissolved in water (0.1 mg/mL). (c) Diffusion of **A5**, **A5** with 5% EDOT-OH, and 10% EDOT-OH in increasing concentrations of CaCl_2_. A smaller radius translates to lower diffusion. (d) Aggregation of **A5** synthesized with varying amounts of PEDOT-OH when dropped into a Ringer solution. Increased PEDOT-OH fraction increases the degree of **A5** aggregation. (e) Micrograph (40× objective) of droplets containing **A5** synthesized with varying amounts of PEDOT-OH when dissolved in water (10 mg/mL). At 50% PEDOT-OH content, the resulting polymer precipitates. (f) MALDI-MS analysis of **A5** with 0, 5, and 10% EDOT-OH added in the synthesis.

Re-examination of the ^1^H-NMR of the EDOT-S monomer (see the Supporting Information), which was used as precursor, did not reveal any EDOT-OH. Furthermore, the very same batch of EDOT-S had been used in the FeCl_3_ methods (Reynolds and Sautter) without yielding a copolymer, suggesting that EDOT-OH is formed in the polymerization reaction and does not result from a pre-existing contamination (Figure 3a).

To investigate whether the presence of EDOT-OH in the polymer contributed to the diffusion behavior, we synthesized the copolymers of EDOT-S with 5 and 10% EDOT-OH using the Okuzaki method. Comparison of the diffusion behavior for these copolymers and **A5** showed that the diffusion rate was lower when there was more EDOT-OH, supporting the hypothesis that the diffusion behavior depends on the copolymeric composition. This was tested by aggregation in Ringer and CaCl_2_ solutions (Figure 3b–f), as a difference in Ringer cast agarose would be difficult to discern. However, Konradsson and Zotti incorporating EDOT-OH still showed diffusion in Ringer cast agarose (Figure 1d). This could be explained by the shorter overall oligomer lengths of Konradsson, and that as seen in the MALDI-MS spectra a smaller amount of EDOT-OH was present using the Fe(OTs)_3_ compared to the persulfate methods. The latter result further supports the hypothesis that increasing the EDOT-OH content decreases diffusion. Increasing the amount of EDOT-OH in the synthesis to >20% gave insoluble macroscopic particles, thus creating an EDOT-S/EDOT-OH mixture, where the EDOT-OH <20 mol % for generating copolymers gives a soluble dispersion (no particles are visible in the light microscope) (Figure 3e). Adding EDOT-OH (10%) in copolymer synthesis slightly reduced the conductivity as compared with **A5** (33 vs 15 S cm^–1^, Table 1).

The high conductivity of **A5** PEDOT-S and S-PEDOT could partly be explained by the presence of sulfuric acid in the polymerization. A plausible explanation for the high conductivity is a combined effect of acidic doping (through sulfuric acid) and oxidative doping (through ammonium persulfate). In addition, the presence of EDOT-OH could generate nanocrystals that are doped during synthesis and that are not available for postdoping with acid. As previously described, we used the Okuzaki protocol—but without the acid—for the synthesis of **A5Y**. The results looked similar in MALDI-MS analysis compared with **A5** but gave a lower conductivity. Dropwise addition of sulfuric acid to **A5Y** postpolymerization did not increase the conductivity; thus, to achieve higher conductivity, acid should be present during synthesis.

To study how the ions alter the electrical properties, we injected **A5** into a Ringer solution to form a hydrogel. The hydrogel was contacted using two Au electrodes, here biased at −0.5 V, and the resulting current was registered. Although biased, we added or removed Ca^2+^ ions from our medium and could detect a corresponding change in the current, where higher Ca^2+^ concentrations resulted in larger currents (lower resistance) (Figure S16). Importantly, the increased conductivity could not be linked to geometrical changes of the **A5** because only modest swelling was seen when moving it to CaCl_2_ solutions (Figure S17). To further investigate these swelling characteristics, **A5** solution (10 mg/mL) was added to isopropanol, which led to the formation of wires (see the Supporting Information). Drying of the wet wire to a dry state resulted in a 25× reduction of the cross-sectional area (see Figure S18). The addition of Ringer solution to the dry wire resulted in a 10× increase of the cross-sectional area forming a hydrogel, and with pure water, the structure was slowly dissolved. This shows that **A5** form hydrogels with >90% water content and that ions are critical for maintaining the hydrogel structure.

**Figure 4:**
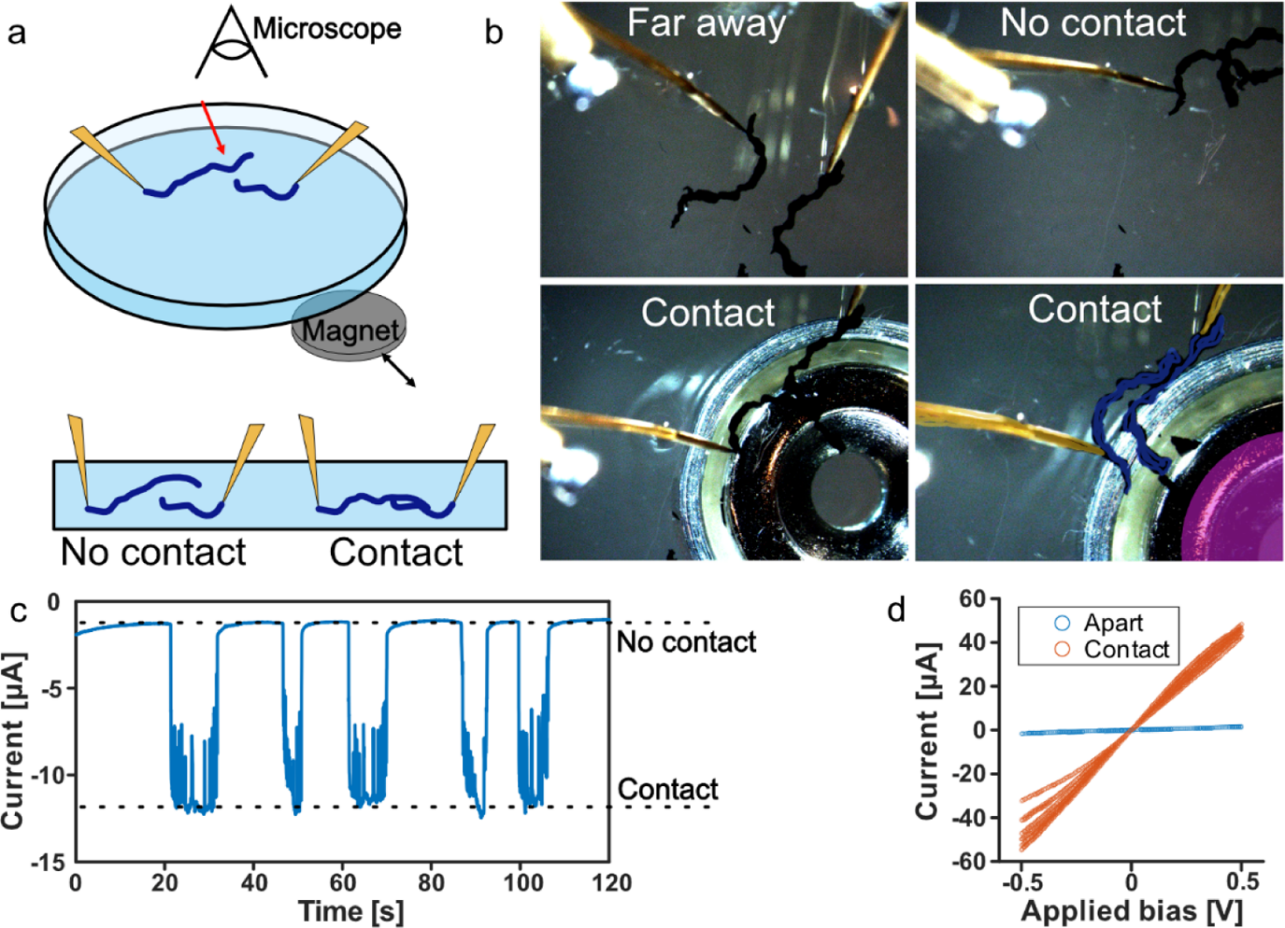
Magnetically actuated **A5** wire switch. (a) Schematic describing the experimental setup: **A5/FeNP** wires (blue) are dispersed in H_2_O and contacted by Au electrodes (yellow). A magnet (gray) is moved underneath the Petri dish containing **A5** and water. The red arrow marks the region, where the **A5** wires make contact. (b) Microscopy images showing the contacted **A5/FeNP** wires being far away from each other, in proximity and in electrical contact when the magnet is underneath. The false-colored overlay shows the electrodes (yellow), **A5** (blue), and magnet (pink). (c) Current flowing between the electrodes (biased at −0.4 V) as the magnet is moved underneath; see movie in the Supporting Information. When the **A5** wires move into contact, a clear increase in current is observed. (d) *C*–*V* measurements between the **A5** wires when electrical contact is on/off.

When optimizing **A5**, one batch was noticed as having a more pronounced aggregation behavior, which could not be completely explained by the content of EDOT-OH monomers in the material. When injected into Ringer solution, this batch (10 mg/mL) formed long threads of an aggregated polymer that could not be reproduced using the 90/10PEDOT-S/-OH material. The increased aggregation behavior of the one batch might be because of incomplete purification. In addition, ICP-AES analysis of the material showed that the **A5** material contains iron, despite an extensive workup with both cation and anion exchanges in the synthesis of the material. The batch that had increased aggregation had a higher content of iron, but there was only a modest difference (1.06 vs 1.03 mg/L (Fe at 238.204 nm)). Based on the average molecular weight of the polymers of approximately 2500–3000 Da, these numbers translate to approximately one Fe-ion (Fe(III) or Fe(II)) per two chains. We then turned our attention to preloading the **A5** with iron (Fe_2_(SO_4_)_3_) as a means to tune the aggregation properties. Mixing **A5** (10 mg/mL) with 2 mM Fe_2_(SO_4_)_3_ or lower concentrations resulted in injectable solutions, but at 5 mM, precipitation occurred (Figure 4c). With 2 mM Fe_2_(SO_4_)_3_, the polymer solution was viscous and challenging to inject. At 1 mM Fe_2_(SO_4_)_3_, the Fe-loaded **A5** solution was still injectable and formed polymer threads when injected into the Ringer solution, which were stable over an extended time period. We also synthesized **A5** without Fe(II) in the polymerization, relying only on persulfate as an oxidant. This reaction proceeded very slowly; therefore, the reaction time was extended to 2 weeks (see the Supporting Information for details). The MALDI-MS measurements (Figure S8) showed a polymer of basically the same size as the others but with an increase in the EDOT-OH content. In the diffusion experiment, it was dissolved completely, and conductivity measurements showed little or no conductivity in the range of the C–H method. Interestingly, the GPC analysis showed *M*_n_ = 3200 and *M*_w_ = 18,000 with a reasonable DPI of 5–6 (Supporting Information), which is indicative that this material is not prone to aggregation as the others (this was also the only material that did not show a time dependency in the GPC analysis) and that incorporation of Fe ions is important in oxidative polymerization to obtain a conductive material. Similar results were previously shown by Konradsson and co-workers.^19^ Adding Fe^3+^ to **A5** postpolymerization resulted in a slight reduction of the conductivity (from 33 to 19 S cm^–1^, Table 1), but clearly added to the beneficial aggregation properties of **A5**.

An interesting feature of **A5** is that it can form hydrogels by extrusion into salt solutions (see the Supporting Information for details). These conductive hydrogels remained stable in pure water over an extended time (>7 months), both in terms of their conductivity and structure. We sought to enable external motion control of this water-stable **A5** to allow for the creation of functional devices such as electrical switches. To realize this, we mixed the **A5** (75 μL, 10 mg/mL) with 5 nm iron nanoparticles (25 μL, 5 mg/mL), followed by extrusion into isopropanol, which resulted in the formation of a several cm long conductive polymer wire. The wire was moved to 0.1 M CaCl_2_ to ensure ionic cross-linking, hence making it water stable. We then moved the formed hydrogel to pure water when performing electrical measurements to minimize the conductivity of the surrounding medium.

Two separate **A5/FeNP** hydrogels were independently contacted using our two electrode setups (Figure 4). The current flow under a low applied bias was monitored while the electrode/(**A5/FeNP**) wires were moved around. When in proximity, albeit without electrical contact, we added a permanent magnet underneath the Petri dish containing the **A5** wires. The magnetic field from the magnet pulls the wires together or pushes them apart, depending on their relative locations. The external magnet induced a high enough magnetic force onto the nanoparticles to move the **A5** wires into electrical contact in a reversible manner. For over 2 min, we moved the **A5** wires into and out of contact five times (see movie in the Supporting Information). When the wires were seen to move together, we observed a corresponding jump to contact in our current reading (Figure 4c). This experiment demonstrates the ability to form magnetically controllable **A5** hydrogels by incorporating Fe_3_O_4_ nanoparticles during extrusion. The addition of 15 wt % of 5 nm magnetic iron nanoparticles (Fe_3_O_4_) to **A5** reduced the conductivity close to 100× (from 33 to 0.39 S cm^–1^, Table 1) when dried films are investigated, which is still much higher than the conductivities seen in tissue.^18^ These reduced conductivities might be related to the poor film-forming properties of the **A5**/Fe_3_O_4_ nanoparticle blend which showed a crackelate and non-homogenous film.

## Conclusions

In conclusion, we have prepared a PEDOT-S derivative (**A5**) that forms water-dispersible nanoparticles that self-aggregate into a highly conductive hydrogel (1–5 S cm^–1^) when injected into a physiologically relevant model. The conductive hydrogel was found to be water stable over many months, and we used its properties to make devices such as magnetoelectric relays. To this end, most of the synthetic methods of PEDOT-S that have been published were reproduced and analyzed using high-resolution MALDI-MS. Surprisingly, only the methods using excess FeCl_3_ as the sole oxidant resulted in the homopolymer PEDOT-S, whereas all the other methods gave a copolymer of P(EDOT-S/EDOT-OH) in different monomer ratios. The formation of the copolymer dictates the aggregation behavior of the PEDOT-S derivatives, which impacts its characteristics to a large extent. Furthermore, all the methods gave polymers of the same length, on average seven to eight monomers long, except for one published method by Konradsson, which gave shorter polymers with an average length of five to six monomers. Thus, three classes of PEDOT-S derivatives with potentially different uses, short and long copolymers and homopolymers, were identified. In general, PEDOT-S derivatives are closer to the definition of oligomers than classical polymers, as also noted by Zotti et al. Comparing the MALDI-MS analysis with GPC was difficult, the latter highly overestimates the length probably because that these oligomers are highly prone to aggregation. For GPC analysis, it is also important to consider the difference between using RI or UV detectors; the former overestimates the polymer weights in the lower range, impacting *M*_n_, but not the higher ones represented by *M*_w_. All the methods used in the polymerization show conductivity values in the same rank order as published in the original publications using two-terminal and four-point probe measurements. One exception was Konradsson’s shorter polymer, which gave a lower conductivity using the two-point measurements, but fall into the rank when a more elaborate film-forming procedure was used along with performing four-point probe characterization. Using no iron catalyst in the reaction still gave a PEDOT-S polymer of the same length as the others, but showed little to no conductivity and little to no tendency to aggregate despite the formation of a copolymer. Incorporation of iron ions between the oligomers seems to be important. **A5**, together with the S-PEDOT published by Okuzaki, displayed the highest conductivities, both about 30 S cm^–1^. This is considerably lower than the published values (1089 S cm^–1^) for S-PEDOT, but no postprocessing optimization was performed. Copolymers were synthesized and evaluated by adding additional amounts of EDOT-OH to EDOT-S in a copolymerization. This generated soluble dispersion of conductive materials up to the point of 20% additions, above which particles started to form, which was not deemed useful for our specific application. We are currently applying these materials, including **A5**, in in vivo studies of conductive materials.

## Supporting Information Available

Details of the synthesis of monomers and polymers; NMR spectra of monomers; details of electrochemical characterization; details of diffusion experiments; MALDI-MS spectra; and GPC data (PDF)
Magnetic manipulation of the **A5/FeNP** wires (AVI)

## Supplementary Material

cm1c04342_si_001.pdf

cm1c04342_si_002.avi
