# Supplementary material for: Method Matters: Exploring Alkoxysulfonate-Functionalized Poly(3,4-ethylenedioxythiophene) and Its Unintentional Self-Aggregating Copolymer toward Injectable Bioelectronics
Source: Chem Mater. 2022 Feb 28;34(6):2752–63. doi: 10.1021/acs.chemmater.1c04342 (PMC8944941; doi:10.1021/acs.chemmater.1c04342)
Supplement: Supplementary file 1 — cm1c04342_si_001.pdf [file cm1c04342_si_001.pdf]

Supporting information

Method Matters: Exploring alkoxysulfonate-functionalized Poly(3,4-ethylenedioxythiophene) and its unintentional self-aggregating copolymer towards injectable bioelectronics

Abdelrazek H. Mousa[a,b‡], David Bliman[a,b‡], Lazaro Hiram Betancourt[c], Karin Hellman[b], Peter Ekström[b], Marios Savvakis[d], Xenofon Strakosas[d], György Marko-Varga[e]

Magnus Berggren[d], Martin Hjort[b], Fredrik Ek[b], and Roger Olsson[a,b*]

[a] Department of Chemistry and Molecular Biology, University of Gothenburg, 405 30 Gothenburg, Sweden. [b] Chemical Biology & Therapeutics, Department of Experimental Medical Science, Lund University, 221 84 Lund, Sweden. [c] Division of Oncology, Department of Clinical Sciences, Lund University, 221 00 Lund, Sweden. [d] Laboratory of Organic Electronics, Department of Science and Technology, Linköping University, 601 74 Norrköping, Sweden. [e] Div. Clinical Protein Science & Imaging, Department of Clinical Sciences and Department of Biomedical Engineering, Lund University, Lund 221 00, Sweden

Table of Contents

Synthesis 3

Synthesis of monomers 3

Polymerization protocols...4

Variations of the A5 method 7

Uv-Vis spectra of polymers 7

High resolution MALDI-MS 9

Electrochemical characterization and diffusion experiment...................................13

NMR spectra of monomers...20

References…………………………………………………………………………22

GPC results………………………………………………………………………..24

Synthesis

All commercial chemicals were used as delivered without further purification. Dry solvents were obtained using an Inert PureSolv MD5 solvent drying system. UV-Vis absorption spectra were measured using Perkin Elmer Lambda 650 and 950 spectrophotometers. ^{1}H- and ^{13}C-NMR were obtained at 400 and 101 MHz, respectively, using a Varian 400/54 spectrometer. Chemical shifts were referenced against solvent residual peaks with methanol-d$_4$ at 3.30 ppm and chloroform at 7.26 ppm.

Synthesis of monomers

Synthesis of sodium 4-((2,3-dihydrothieno[3,4-b][1,4]dioxin-2-yl)methoxy)butane-1-sulfonate, sodium salt (EDOT-S)

EDOT-S was synthesized according to the previously published procedure with a slight modification[1], NaH (0.126 g, 3.14 mmol, 1.2 equiv.) was added to a solution of EDOT-OH (0.451 g, 2.62 mmol) in 8 mL dry THF in a microwave vial under a nitrogen atmosphere. The mixture was stirred at room temperature for 20 min then cooled to 0 ^{o}C using an ice bath where (0.35 mL, 3.43 mmol, 1.3 equiv.) 1,4-butane sultone was added dropwise. The ice bath was removed and the reaction was allowed to reach room temperature then heated to 65 ^{o}C overnight where a pale orange solution was formed. After cooling to the room temperature, an orange colored gel was formed which was dissolved by adding 10 mL MeOH. Et_2O was added resulting in formation of a white precipitate. The precipitate was collected by centrifugation at 4500 rpm for 10 minutes. This is repeated three times then the obtained solids were dried using the high vacuum line giving off white solids 0.824 g (95 % yield). ^{1}H NMR (400 MHz, CD_3OD) δ 6.37 – 6.34 (m, 2H), 4.30 – 4.20 (m, 2H), 4.04 – 3.97 (m, 1H), 3.71 – 3.59 (m, 2H), 3.54 (t, J = 6.3 Hz, 2H), 2.87 – 2.80 (m, 2H), 1.91 – 1.82 (m, 2H), 1.76 – 1.66 (m, 2H). ^{13}C{^{1}H} NMR (101 MHz, CD_3OD) δ 143.03, 142.97, 100.35, 100.25, 74.12, 72.34, 70.25, 67.23, 52.36, 29.67, 22.92.

Synthesis of sodium 4-((5,7-dibromo-2,3-dihydrothieno[3,4-b][1,4]dioxin-2-yl)methoxy)butane-1-sulfonate (diBrEDOT-S)

diBrEDOT-S was synthesized following the literature procedure with a slight modification.[2] To a solution of EDOT-S (0.220 g, 0.66 mmol) in 4 ml DMF under an inert atmosphere of nitrogen, a solution of NBS (0.273 g, 1.53 mmol, 2.3 equiv.) in 4 ml DMF was slowly added at 0 ^{o}C, then the reaction was kept stirring at room temperature for 24 h. The color of the solution was bright orange. The product was transferred into two centrifuge tubes then 45 ml acetone was added to each tube causing immediate precipitation. The tubes were centrifuged for 5 minutes at 4500 rpm two times until clear mother layer was obtained. The obtained off white solids were dried using the high vacuum line giving 0.144 g (44 % yield). ^{1}H NMR (400 MHz, CD_3OD) δ 4.41 – 4.32 (m, 2H), 4.16 – 4.07 (m, 1H), 3.77 – 3.64 (m, 2H), 3.56 (t, J = 6.2 Hz, 2H), 2.89 – 2.78 (m, 2H), 1.95 – 1.80 (m, 2H), 1.78 – 1.67 (m, 2H). ^{13}C{^{1}H} NMR

(101 MHz, CD_3OD) δ 141.3 , 141.29 , 85.80 , 85.78 , 74.9 , 72.5 , 69.8 , 67.6 , 52.4 , 29.7 , 22.9. This compound is thermally unstable and was used directly.

Sodium 4-((2,3-dihydrothieno[3,4-b][1,4]dioxin-2-yl)methoxy)butane-2-sulfonate (S-EDOT)

NaH (60 % in mineral oil, 488 mg, 12.2 mmol) was added to a solution of EDOT-OH (2.00g, 11.6 mmol) in dry THF (40 mL) in a 250 ml round flask under nitrogen. The reaction mixture was stirred under nitrogen at r.t. for 20 min, then cooled on ice and 2,4-butanesultone (1.6 mL, 15.4 mmol) was added dropwise. The ice bath was removed, the reaction was allowed to reach r.t. and was then heated to 70 °C for 16.5 h. The reaction was then allowed to reach r.t. and quenched with MeOH (60 mL). The mixture was poured in to Et_2O (700 ml) resulting in a precipitation. The precipitate was collected by filtration and washed repeatedly with DEE (5x100 mL), dried by water aspiration in the funnel followed by further drying on oil pump resulting in an off white solid (2.44g, 64%). 1H-NMR (D_2O) matches the published data.[3] The integral for the thiophene protons is low, possibly due to H/D exchange. ^1H NMR (400 MHz, CD_3OD) δ 6.56-6.46 (m, app. 1H), 4.45-4.39 (m, 1H), 4.28 (ddd, J = 11.9, 2.3, 1.0 Hz, 1H), 4.10 (dd, J = 12.0, 6.9 Hz, 1H), 3.82-3.62 (m,4H), 3.06-2.91 (m, 1H), 2.82-2.16 (m, 1H), 1.74-1.60 (m, 1H), 1.29 (d, J = 6.9 Hz, 3H).

Polymerization protocols

PEDOT-S using Konradsson's method

PEDOT-S was prepared following the literature procedure reported by Konradsson[5] with slight modification. A solution of $K_2S_2O_8$ (0.327 g, 1.22 mmol) and $FeCl_3$ (0.005 g, 0.031 mmol) in water (3 mL) was added dropwise to a stirred solution of EDOT-S (0.200 g, 0.61 mmol) in water (3 mL). The reaction mixture was stirred for 3 h at rt then the reaction was quenched by adding 40 mL of acetone causing precipitation of a deep blue colored solid which was collected after centrifugation for 5 minutes at 4500 rpm. The collected precipitate was dissolved in water (7 mL) and precipitated again with acetone (40 mL) followed by centrifugation. The procedure was repeated three times . The obtained precipitate was dissolved in 10 ml water and was distributed into five Vivaspin® concentrator tubes (3000 MWCO) followed by centrifugation for 10-15 minutes at 4500 rpm . This was repeated three times then the collected concentrate was freeze-dried giving a deep blue soft material. Yield: 147 mg (73 % with respect to monomer unit). The polymer was characterized using UV-Vis spectroscopy and MALDI-MS.

PEDOT-S using Zotti's method

PEDOT-S was prepared following the literature procedures reported by Zotti[6], A solution of EDOT-S (0.08 g, 0.24 mmol) in a degassed water (2.4 mL) was heated to 80 °C then $Fe(OTs)_3$. $6H_2O$ (0.49 g, 0.72 mmol) was added. The color of the solution immediately turned dark blue. The reaction mixture was kept stirring at 80 °C for 3.5 h then was cooled and filtered using a glass frit filter. The filtrate was purified using cation and anion exchange resins where 10 g of Letwatit MonoPlus S108 and 10 g of Lewatit MP62 were used subsequently to get rid of the

undesired ions. Filtration offered a deep blue solution which was freeze-dried. Yield: 36.4 mg (45 % with respect to monomer unit). The polymer was characterized using UV-Vis spectroscopy and MALDI-MS.

PEDOT-S using Reynold's method

PEDOT-S was prepared following the literature procedures reported by Reynolds with a slight modification[7], a mixture of EDOT-S (0.1 g, 0.3 mmol) and $FeCl_3$ (0.162 g, 3.3 mmol, 3.3 equiv.) were dissolved in 15 mL of chloroform. The reaction was kept stirring at room temperature for 24 h under an inert atmosphere of nitrogen. Then 100 mL of methanol was added to the obtained solution causing precipitation of dark solids, which were isolated through centrifugation. The dark precipitate was stirred in a 1 M sodium hydroxide solution in methanol (200 mL) for 48 hours. The solution was filtered, and the black polymer powder was stirred in 200 mL of deionized water for 24 hours and then filtered again giving a deep purple solution. Addition of acetone to precipitate the polymer was not successful and instead the solvents was evaporated using the Rotvap giving dark purple solids. Finally, the polymer was dialyzed with deionized water for 3 days using a 3500 g/mol cutoff membrane and change in the color to a blue was observed. The solution obtained after dialysis was freeze-dried giving a fluffy blue material. Yield: 25 mg (25 % with respect to monomer unit). The polymer was characterized using UV-Vis spectroscopy and MALDI-MS.

PEDOT-S using the Sautter method

To a solution of the EDOT-S (0.248 g, 0.75 mmol) in 9 ml MilliQ water under an inert atmosphere of nitrogen, $FeCl_3$ (0.486 g, 3 mmol) was added then the solution was stirred at room temperature for 8 h then the temperature was increased to 100 ºC and the reaction was kept for 3 h at this temperature. After cooling down to room temperature, cation and anion exchange was used as a purification method to get rid of the undesired cations and anions formed during the polymerization reaction. In details, 4.5 g of the cation exchange resin (Lewatit Monoplus S108H) was first washed with water combined with 4.5 g of the anion exchange resin (MP62WS, Lanxess) previously washed with water then added to the crude polymer and left stirring for 4 hours. The cation and the anion exchange resins were removed by filtration and the filtrate was freeze-dried giving a dark blue fluffy material. Yield: 150 mg (60 % with respect to monomer unit).

PEDOT-S using C-H arylation method

1 mL anhydrous DMF was added to a mixture of EDOT-S (0.05 g, 0.15 mmol), compound **2** (0.073 g, 0.15 mmol), $P(p\text{-tolyl})_3$ (0.004 g, 0.015 mmol), and Cs_2CO_3 (0.102 g, 0.31 mmol) under an inert atmosphere of nitrogen in a 5 ml microwave vial then 0.25 ml of a stock solution of $Pd(OAc)_2$ in DMF corresponding to (0.002 g, 0.007 mmol) was added to the vial. The reaction mixture was stirred for 48 h at 100 °C. After cooling, the mixture was quenched with MilliQ water (2 mL) followed by addition of excess acetone. The resulting suspension was centrifuged (5 min, 5000 rpm). The precipitate was dissolved in water (2 mL) and again

precipitated from acetone. This procedure continued until a clear solution was obtained. Finally, the precipitate was dried under vacuum to yield the PEDOT-S polymer as a brown solid in 30 % yield.

PEDOT-S using the A1 method

To a mixture of EDOT-S (50 mg, 0.15 mmol) and $FeSO_4.7H_2O$ (25 mg, 0.09 mmol, 0.6 equiv.), 4 ml of 1M aq. H_2SO_4 was added under an inert atmosphere of nitrogen. $(NH_4)_2S_2O_8$ was dissolved in 1 ml MilliQ water giving a clear viscous solution which was added dropwise to the above solution giving a blue solution within 15 seconds. The reaction was stirred for 20 h at r.t. The crude was purified using a pre-washed cation and anion exchange resins. For cation exchange, the crude was added to 2.35 g Lewatit MonoPlus S108 ion exchanger resin and was stirred for 2h. Vacuum filtration was carried out using an empty 10g biotage column and 3 ml water was used to wash the cation exchange resin. The blue colored filtrate was subjected to anion exchange where 3.12 g Lewatit MP62 ion exchanger resin was added followed by stirring for 2 h. The anion exchange resin was filtered out and a dark blue solution was obtained. The solution was freeze-dried giving a fluffy dark blue material contaminated with white solid so the ion exchange workup was repeated again to get rid of the impurity and a deep blue soft material was obtained. 18 mg was obtained after the freeze-drying.

A5 with EDOT-OH

The polymerization was carried out following the A5 method described in the main article varying the mol ratio of EDOT-S to EDOT-OH. The yield (mass %) for PEDOT-S/PEDOT-OH copolymer was 73%, both in the case of 5 and 10 % EDOT-OH.

S-PEDOT using Okuzaki's method

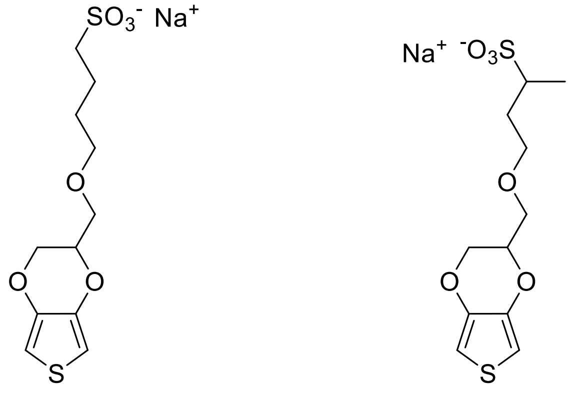

1 **2**

Figure S1. EDOT-S (**1**) used in this work to make PEDOT-S and S-EDOT (**2**) used by Okuzaki et al.

S-PEDOT was synthesized following the procedure for A5 using S-EDOT (100 mg, 0.303 mmol), $FeSO_4*7H_2O$ (0.6 eq., 50 mg, 0.180 mmol) in 1M H_2SO_4 (1.7mL) and $(NH_4)_2S_2O_8$ (2

eq., 138 mg, 0.605 mmol) dissolved in 0.3ml of MQ water. Isolated as a deep blue solid after freeze drying (77 mg, 77%).

Variations of the A5 method

These polymerizations were carried out following the A5 method with the modifications specified below. The workup was carried out as described for the Sautter procedure.

A5Y method

The polymerizations were carried out following the A5 method with water as solvent in place of 1M H_2SO_4 with 0, 10, 20 and 30% EDOT-OH. The yields calculated as mass recovery based on monomer were 78%, 80%, 81% and 79%, respectively.

A5 synthesis without addition of Iron (A5sFe)

The polymerization was carried out as A5 excluding $Fe(II)SO_4*7H_2O$ and leaving the reaction for 14 days at room temperature and the yield calculated as mass recovery based on monomer was 38%.

Uv-Vis spectra of polymers

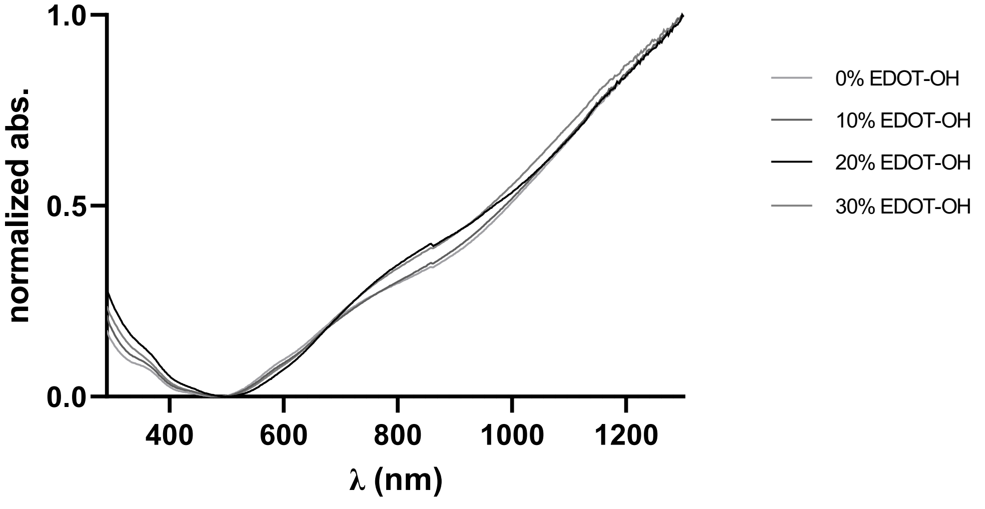

Figure S2. UV-Vis spectra of **A5Y** with 0-30% EDOT-OH.

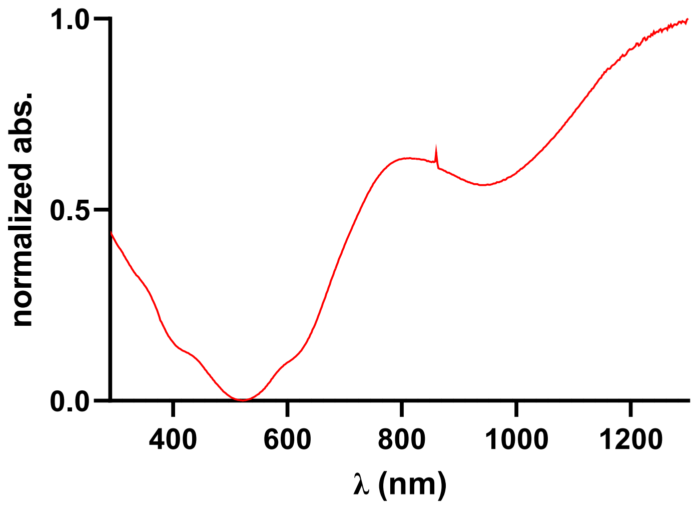

Figure S3. UV-Vis spectra of A5sFe.

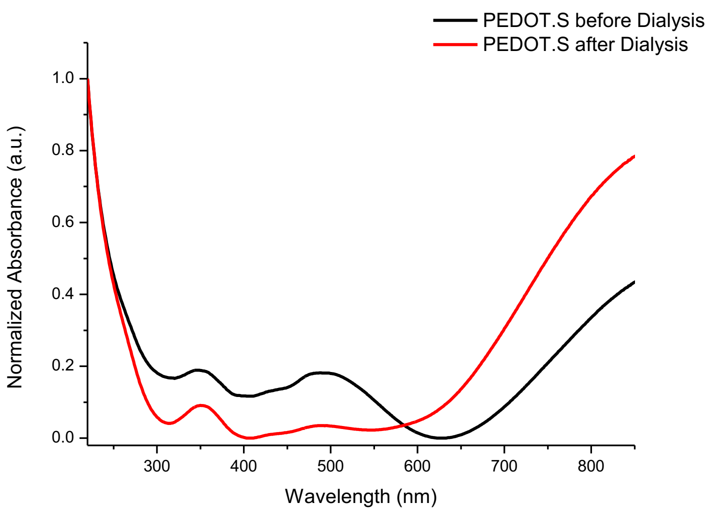

Figure S4. UV-Vis spectra of Reynolds polymer before and after dialysis.

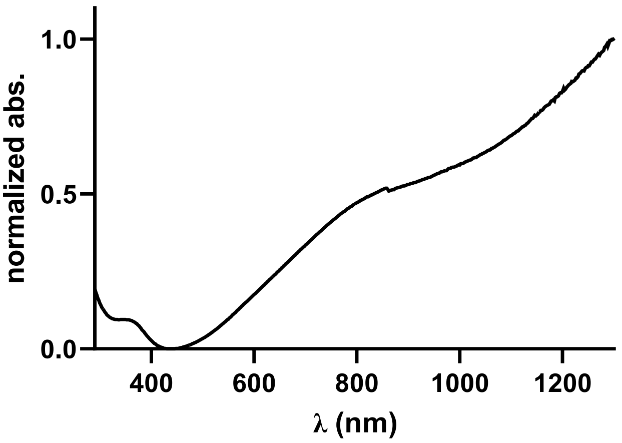

Figure S5. UV-Vis spectrum of S-PEDOT (Okuzaki).

High resolution MALDI-MS

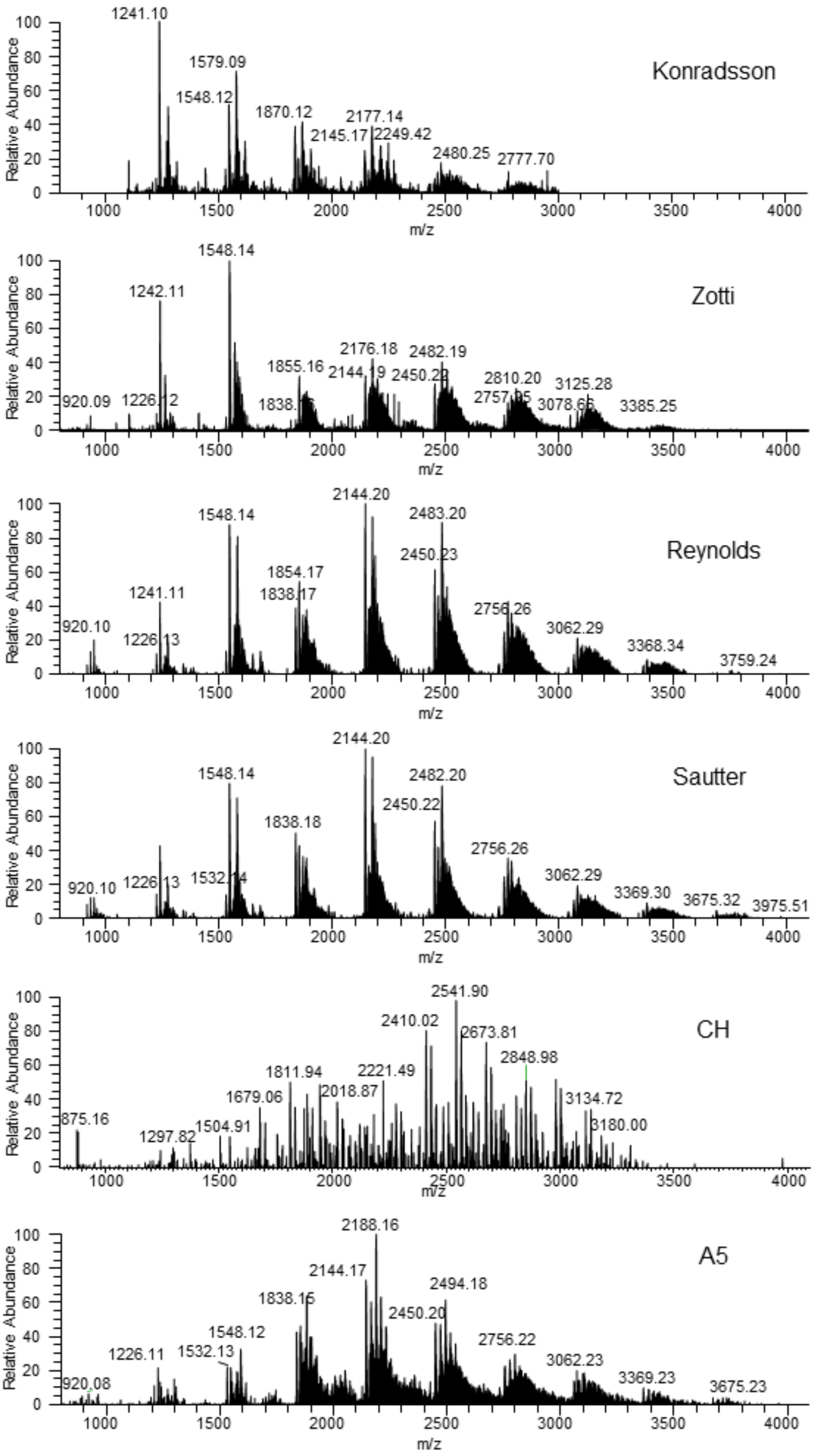

Figure S6. High resolution MALDI-MS spectra for PEDOT-S synthesized by different methods.

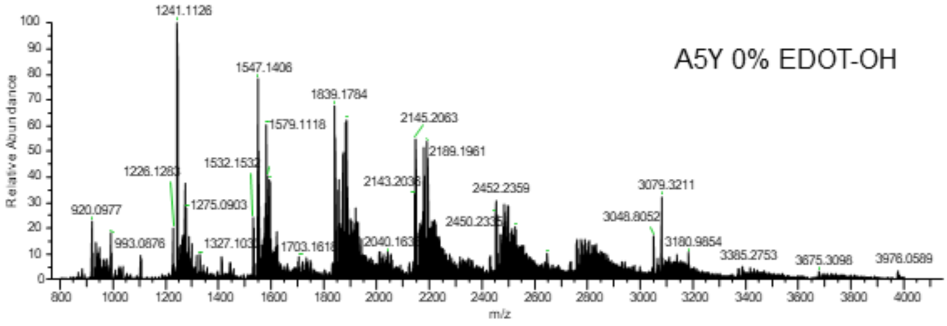

Figure S7. High resolution MALDI-MS spectra of PEDOT-S synthesized by the **A5Y** method.

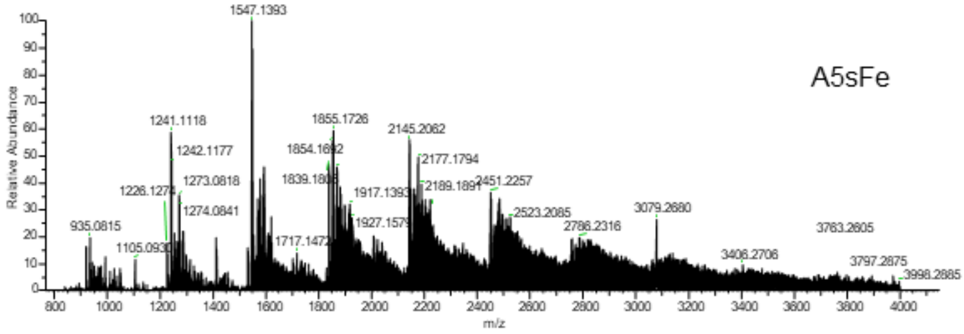

Figure S8. High resolution MALDI-MS spectra of PEDOT-S synthesized as **A5** but excluding Iron catalyst (**A5sFe**).

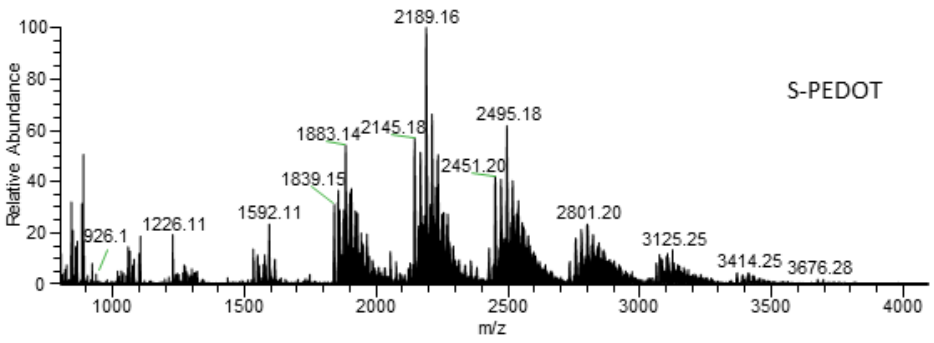

Figure S9. High resolution MALDI-MS spectra of S-PEDOT.

Dynamic Light Scattering (DLS)

DLS measurements of **A5** was carried out using a Malvern Zetasizer Nano instrument with disposable cuvettes at 25 °C. All cuvettes and vials were rinsed three times with filtered (0.2 µm PVDF filter) 1 mM NaCl solution before use. **A5** was dissolved in prefiltered (0.2 µm PVDF) 1 mM NaCl solution and ultrasonicated. The solution was then filtered (0.2 µm PVDF) before analysis.

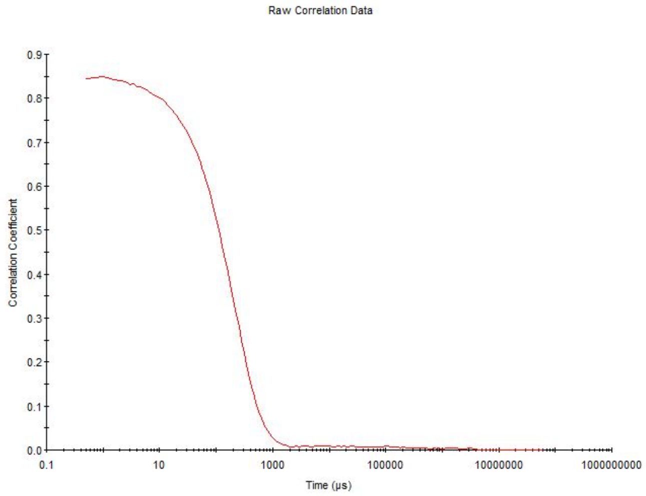

Figure S10. Correlation curve for A5 in 1mM NaCl (aq.).

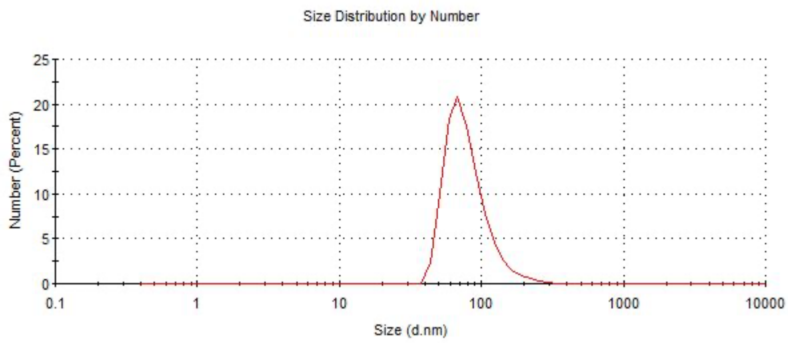

Figure S11. Size distribution of **A5** weighted by number.

Electrochemical characterization and diffusion experiment

Photo of diffusion experiment.

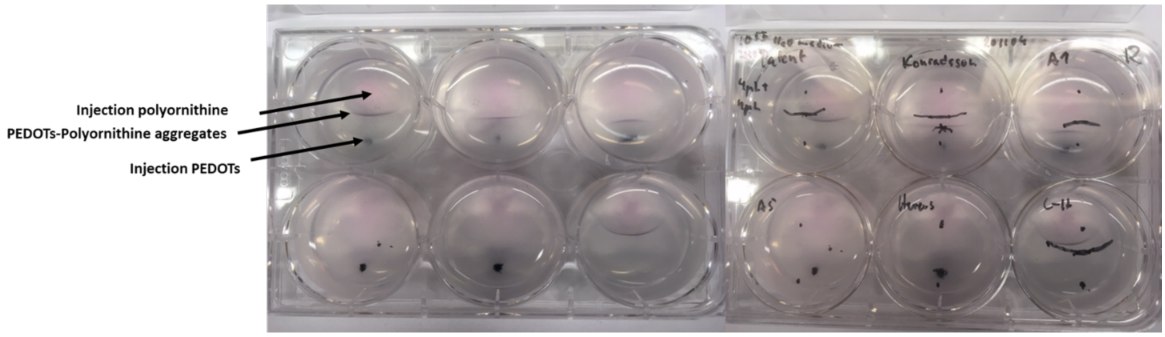

Figure S12. Diffusion experiments in agarose 0.5% (Ringer solution pH 7.2) using polyornithine and PEDOT-S.

Electrical measurements of aggregated A5

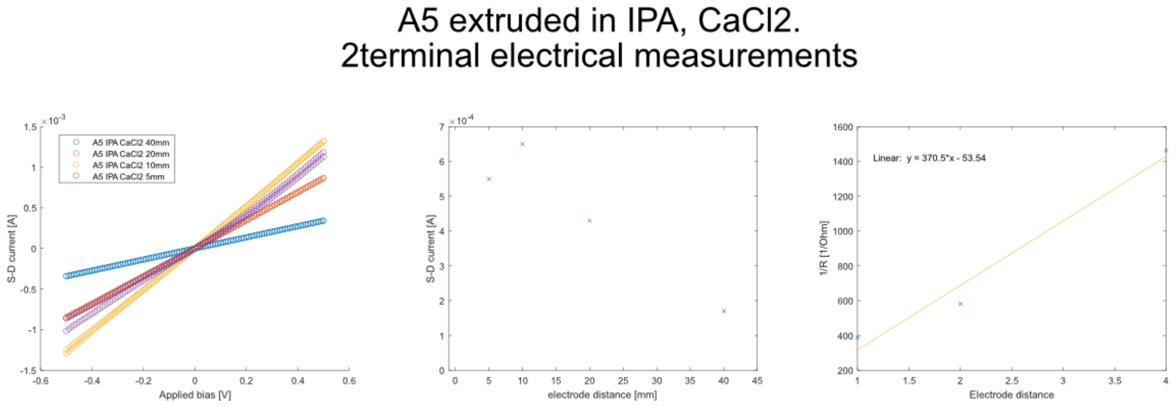

Figure S13. A5 extruded into isopropanol, followed by making it water stable in CaCl2. Current-voltage relationship was mapped at different distances spanning several cm while being dispersed in MQ-water. At 5mm electrode distance, it was challenging to establish a

good contact between electrode and A5. The transmission line model was used to extract the conductivity of the A5 to be about 30Scm^{-1} (assuming the A5 to be a 100um diameter cylinder).

Electrical measurement of A5 in agarose

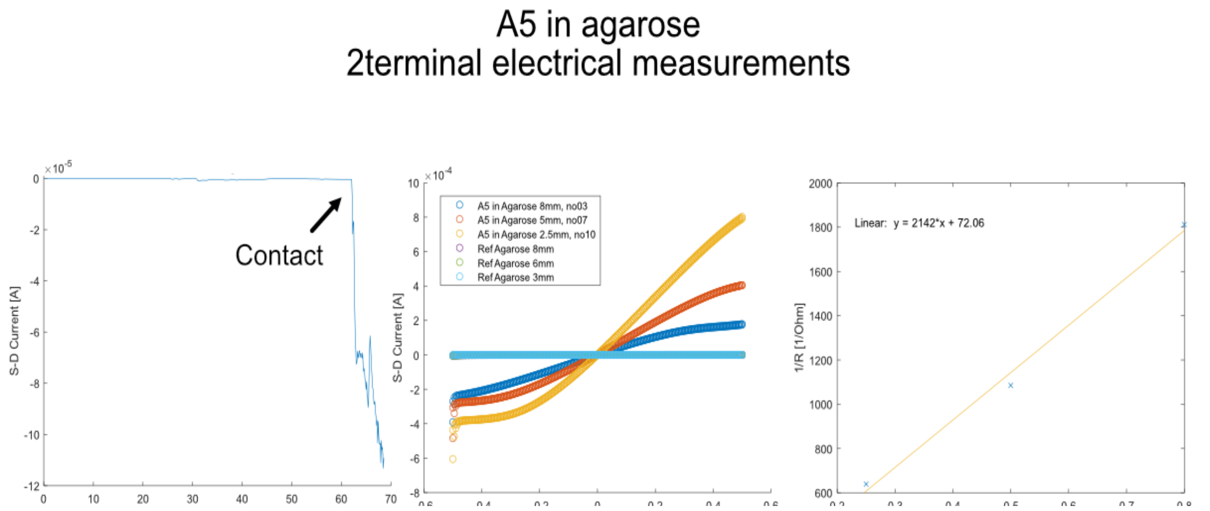

Figure S14. A5 injected into agarose and measured using a 2 terminal setup. (a) shows the current flowing in between the two electrodes while trying to make contact with the A5. Upon poking the A5 with the electrodes, an obvious jump to contact can be seen. Applied bias: -0.5V. (b) Current-voltage sweeps at different A5 distances. At voltages larger than about +-0.2V, the characteristics deviate from linear behavior. (c) transmission line model to extract a conductivity of 5 Scm^{-1} when assuming an A5 geometry of a 100um diameter cylinder.

Long term A5 water stability

A5 wires largely retained their shape and conductive properties even after months in solution. We extruded **A5** into low concentration $CaCl_2$ (0.01M) which is close to the limit where we see the minimal aggregation radius. Using a two terminal setup, we verified the conductive nature of the extruded hydrogel followed by long term storage in MQ water. Storing in water presents a worst-case scenario where we expect drainage of salts and small polymer entities from the hydrogel leading to reduced conductivity and possible collapse of the formed structure. Even after 6 months in water, the hydrogel retained shape without any signs of polymer diffusion. The electrical resistance of the hydrogel was seen to increase somewhat over the 6 months period, but was still much lower than the surrounding medium (Figure S15). We hypothesize that the increased resistance is due to a loss of small polymer entities which contribute to the conductive properties without affecting the overall shape of the hydrogel.

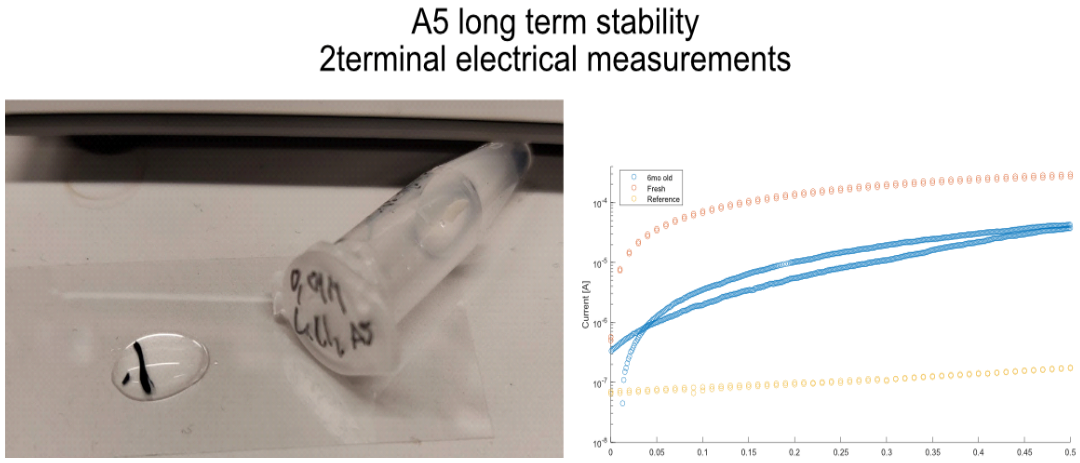

Figure S15. Cross-linked A5 hydrogel long term stability in MilliQ-water.

A5 was extruded into 0.01M $CaCl_2$ to form conductive wires. When freshly made, currents in the order of 0.1mA were observed when applying 0.5V. After 6 months storage in MilliQ-water, the wire still retained it's shape and was still conductive. Storing in MilliQ-water presents a worst case scenario since this is expected to wash away some of the cations that enable the **A5** to aggregate.

A5 with more or less calcium ions

A5 aggregates can be loaded up with varying amounts of calcium ions which can be used to tune the electrical properties. In this experiment, **A5** was injected into Ringer solution to form aggregated wires which were moved to a droplet containing 200µL fresh Ringer, see Figure S16. The **A5** was contacted using two Au coated W electrodes and a potential difference of -0.5V was applied using a Keithley. The resulting current was measured over time. Drops of 2M $CaCl_2$ or 0.5M EDTA was added to either increase or decrease the amount of Ca^{2+} in solution.

We observed a stepwise increase of the current (decrease of the resistance) when more Ca^{2+} was added. EDTA is known to bind calcium and we could see a stepwise decrease of the current (increase of resistance) as we added more EDTA. Interestingly, we observe that adding the same molar amount of EDTA as $CaCl_2$, brings back the current close to the original levels.

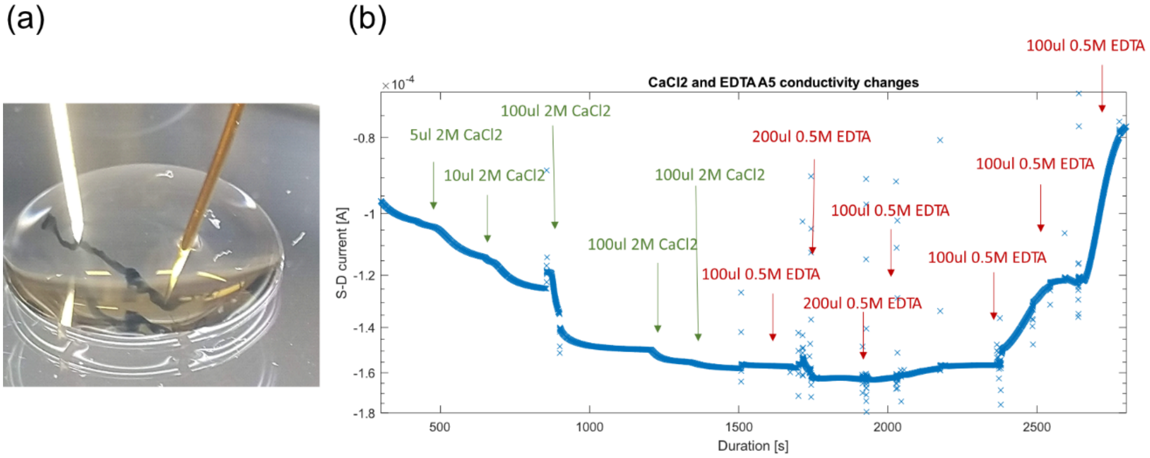

Figure S16. (a) Photograph depicting the contacted **A5** wire as it is dispersed in Ringer buffer. Contacts were not moved for the duration of the experiment. (b) Current flowing in between the electrodes in (a) during the experiment. $U_{applied}$=-0.5V. Addition of $CaCl_2$ solution is shown in green text while addition of EDTA is shown in red. Reference currents in the medium when the **A5** was not contacted were about 1000x lower.

A5 swelling in $CaCl_2$

Extruding **A5** into isopropanol (IPA) provides a quick and straight forward way to retrieve conductive wires. However, in order to make these wires water stable, ions such as Ca^{2+} need to be added. In the calcium dosing experiment presented above, it became apparent that the ionicity of the medium could be used to tune the resistance of the **A5** wire. Here, we set out to analyze whether this could stem from a physical change in size of the **A5**. We added a 10µL droplet of **A5** [10mg/mL] to IPA and let it form a thin disk that could be pushed around using a tweezer. We measured the diameter of the disk, followed by aspiration of the IPA and addition of 0.1M $CaCl_2$. After 6 min, we remeasured the diameter of the disk and found that it was slightly larger. After this, we aspirated the $CaCl_2$ and again added IPA instead. After 6 min, we measured the diameter of the disk and yet again found that it forms a smaller structure in IPA compared to the $CaCl_2$. Repeated cycling rendered the same result, see Figure S17.

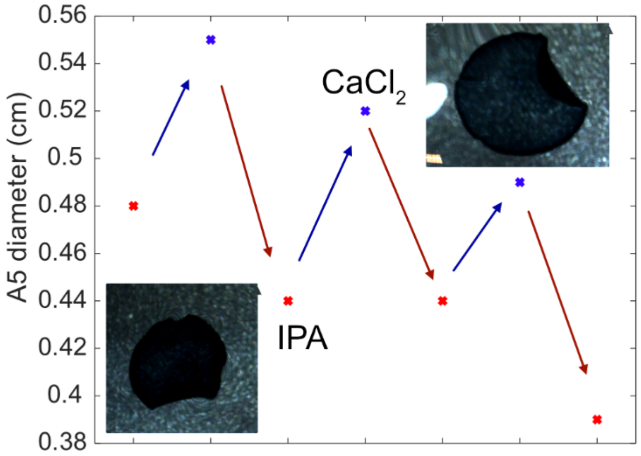

Figure S17. A5 swelling in IPA/$CaCl_2$. A thin **A5** disk was sequentially submerged in IPA or 0.1M $CaCl_2$ and the diameter of the disk was recorded. When submerged in $CaCl_2$, the disk had a larger diameter. Top/bottom micrograph depicts the **A5** in $CaCl_2$/IPA.

In an attempt to investigate the water content of the **A5** wires, we formed the wires in isopropanol, Figure S18. Drying the wire in air led to a 25x reduction in cross section. Rehydrating the dried wires in Ringer solution or in water made it swell again.

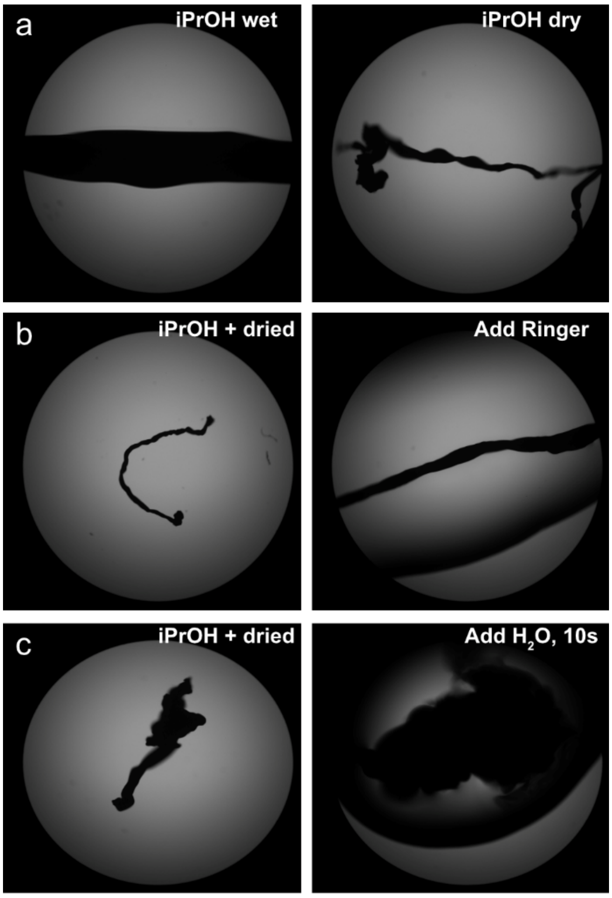

Figure S18. Swelling properties of A5 wires; A**,** the drying of the wet wire to a dry state resulted in a 25x reduction of the cross-section area. B, The addition of Ringer solution to the dry wire resulted in a 10x increase of the cross-section area forming a hydrogel, C, and with pure water, the structure was slowly dissolved.

Four point probe and four-line-probe electrochemical characterization

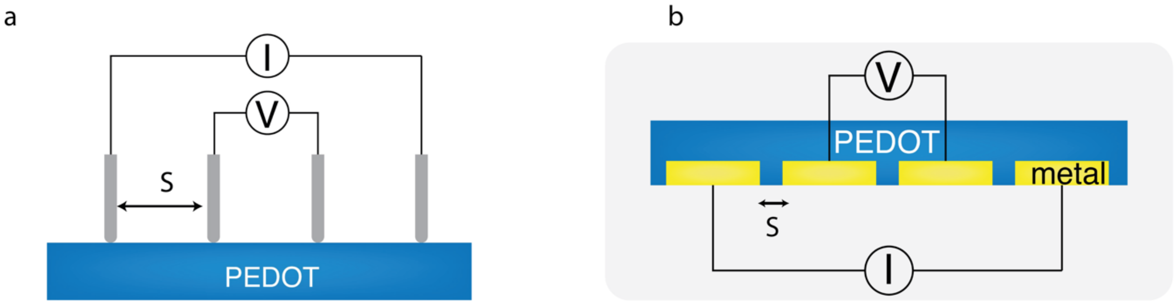

Figure S19. The different setups for conductivity measurements a) Schematic of a four-point-probe setup with S ~ 1mm. b) Schematic of a four-line-probe setup with Au patterned lines and S = 15 μm.

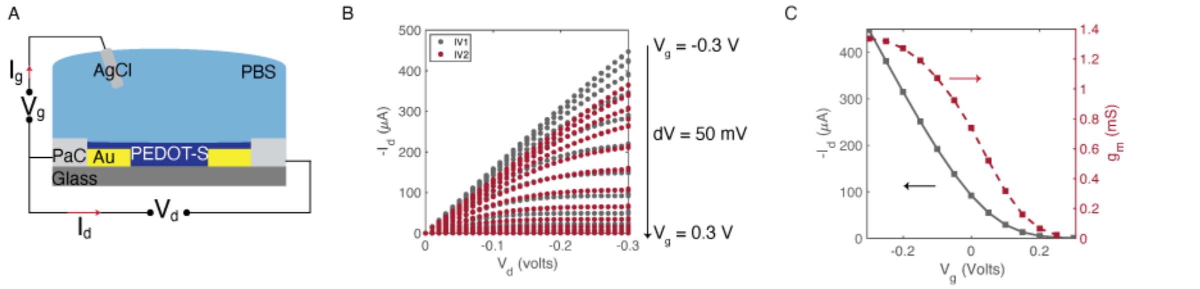

Figure S20. A5 as channel on OECTs. (a) Schematic of an OECT with A5 as a channel and AgCl as a gate. The gate and the channel are immersed in a NaCl 0.1 M electrolyte. (b) An IV curve of the OECT. Upon application of an increased gate voltage the channel is being de-doped and the drain current decreases. (c) Calculated transfer curve and transconductance from an IV curve with $V_d = 0.3$ V.

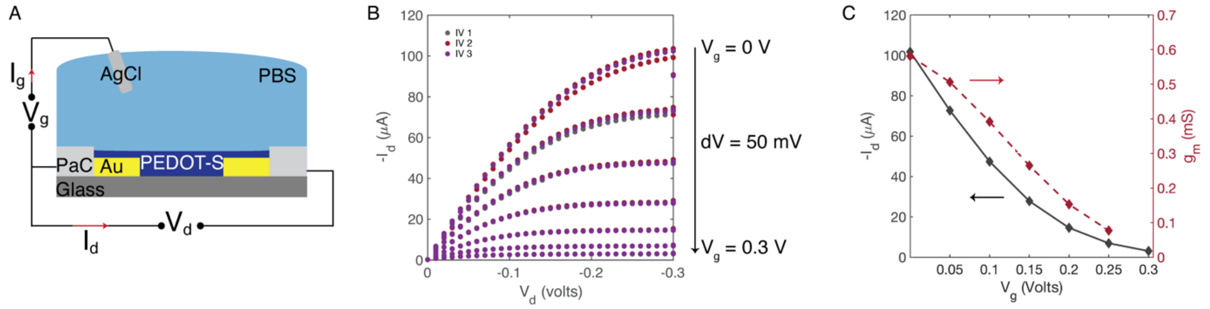

Figure S21. A5 OECT channel made from a 10 mg/mL A5 solution in 1 mM Fe^{3+} . (a) Schematic of an OECT with **A5** as a channel and AgCl as a gate. The gate and the channel are immersed in a NaCl 0.1 M electrolyte. (b) An IV curve of the OECT. Upon application of an increased gate voltage the channel is being de-doped and the drain current decreases. (c) Calculated transfer curve and transconductance from an IV curve with $V_d = 0.3$ V. The highest transconductance is at $V_g = 0$ V. The channels exhibited typical OECT behavior as shown from the IV with stable performance in aqueous environments. The peak transconductance is at $V_g = 0$V with a value of 0.6 mS. This means that the highest amplification properties of the OECTs are at zero gate voltage. This is beneficial for interfacing the OECTs with measurement units where no additional V_g is needed to be applied.[8]

NMR spectra of monomers

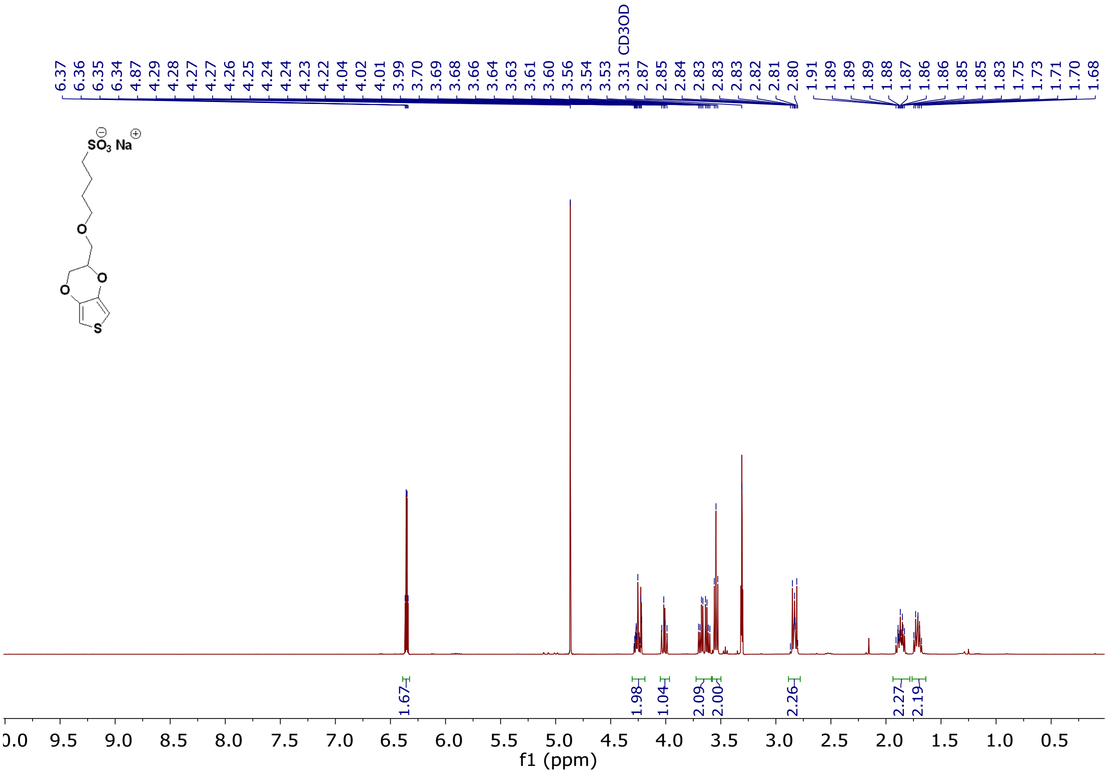

Figure S22. ^{1}H NMR spectrum (400 MHz, CD_3OD) of the EDOT-S.

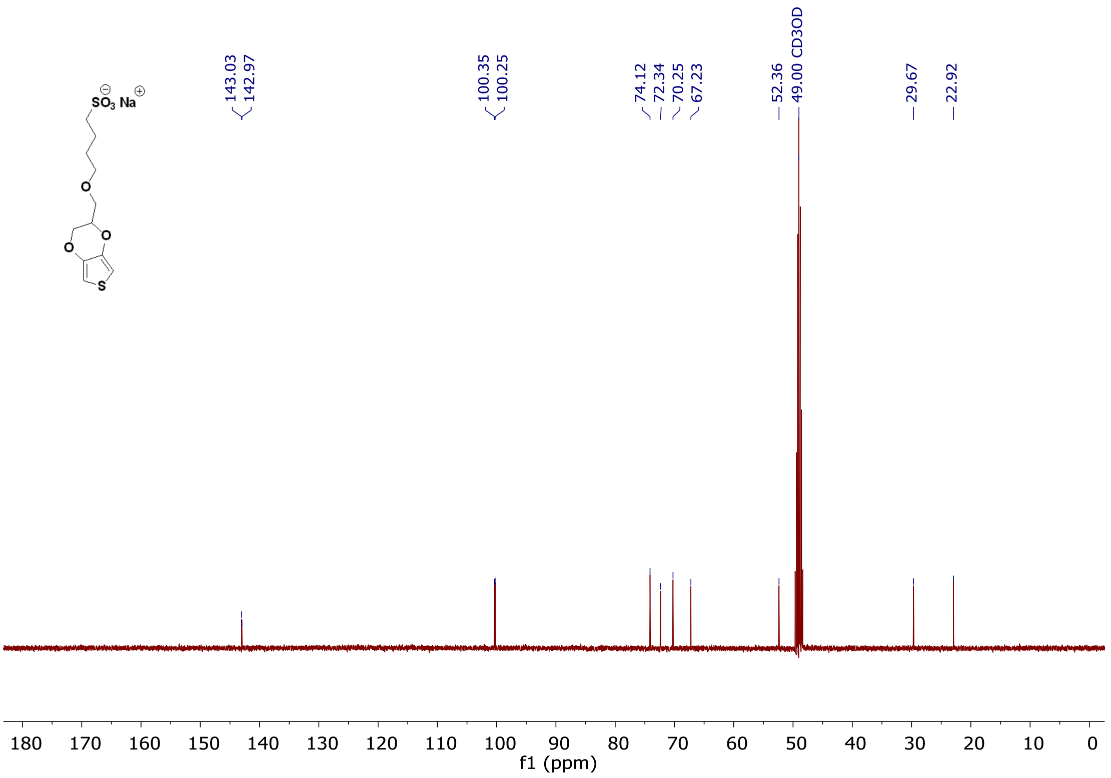

Figure S23. $^{13}C\{^1H\}$ NMR spectrum (101 MHz, CD_3OD) of the EDOT-S.

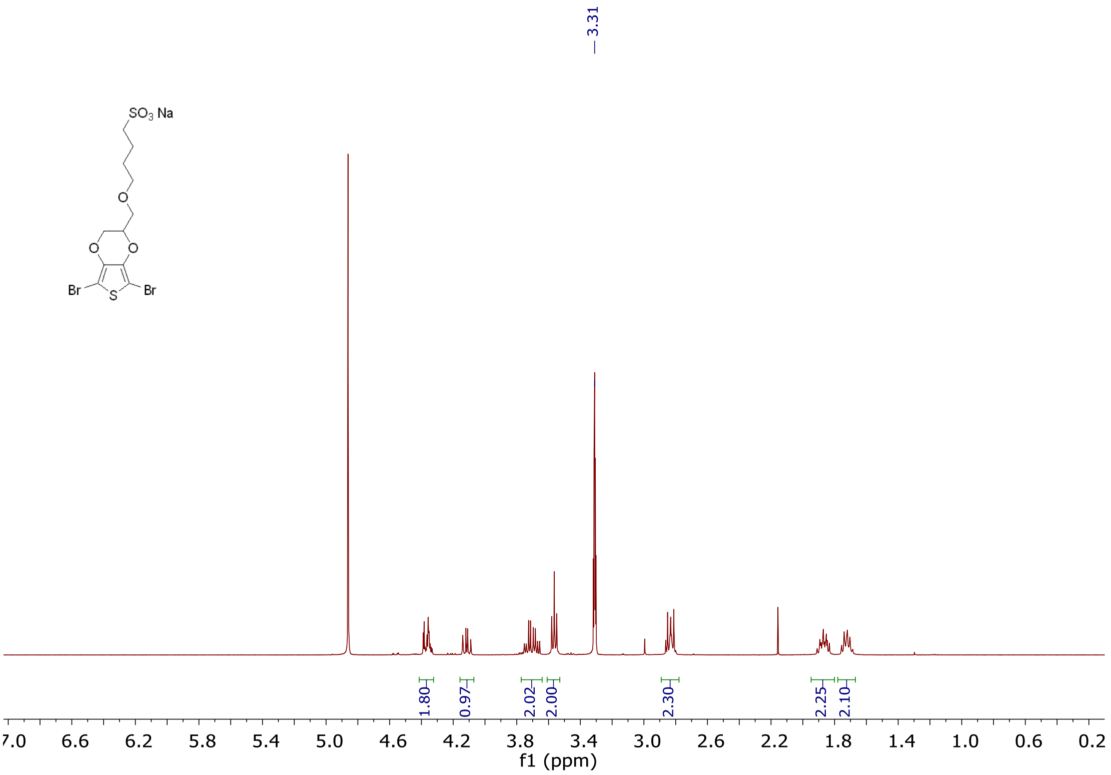

Figure S24. ^{1}H NMR spectrum (400 MHz, CD_3OD) of 4-((5,7-dibromo-2,3-dihydrothieno[3,4-b][1,4]dioxin-2-yl)methoxy)butane-1-sulfonic acid, sodium salt (diBrEDOT-S).

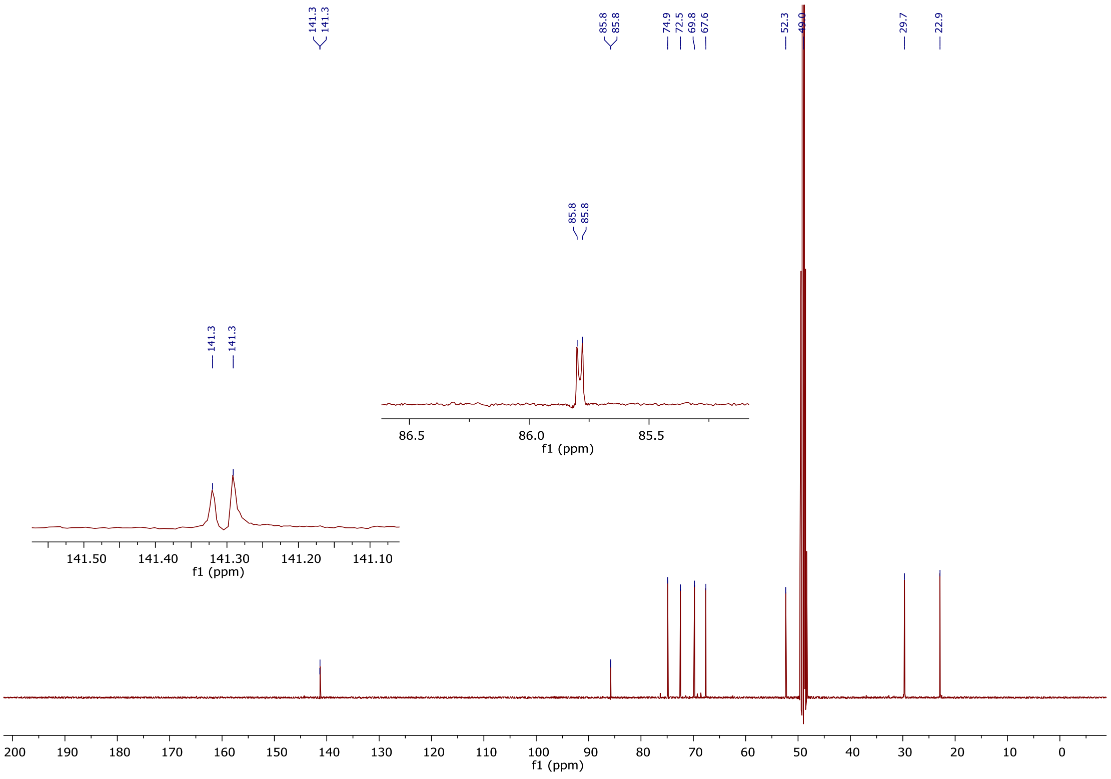

Figure S25. $^{13}C\{^1H\}$ NMR spectrum (101 MHz, CD_3OD) of 4-((5,7-dibromo-2,3-dihydrothieno[3,4-b][1,4]dioxin-2-yl)methoxy)butane-1-sulfonic acid, sodium salt (diBrEDOT-S).

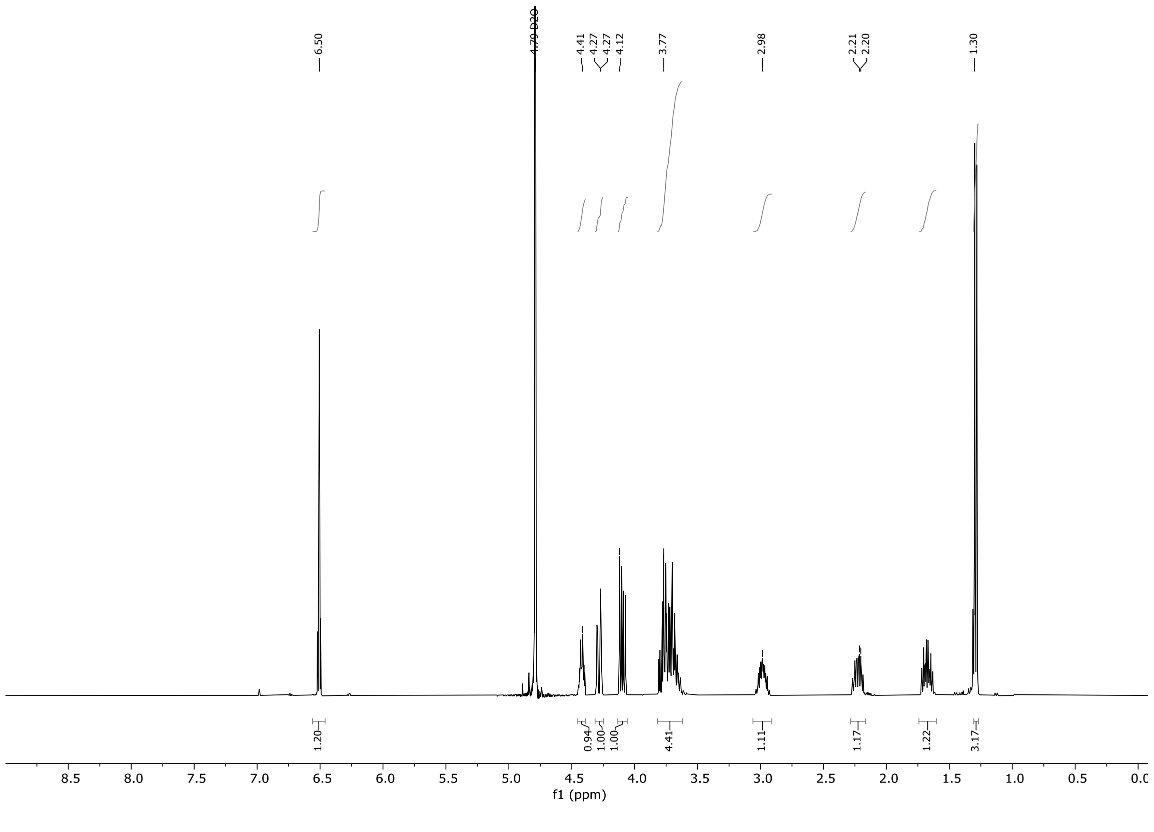

Figure S26. ^1H NMR spectrum (400 MHz, D_2O) S-EDOT.

References

1. Stéphan, O.; Schottland, P.; Le Gall, P.-Y.; Chevrot, C.; Mariet, C.; Carrier, M., Electrochemical behaviour of 3, 4-ethylenedioxythiophene functionalized by a sulphonate group. Application to the preparation of poly(3, 4-ethylenedioxythiophene) having permanent cation-exchange properties. *J. Electroanal. Chem.* **1998,** *443,* 217-226.
2. Yu, H.-h.; Ayalew, H.; Wang, T.-l.; Wang, T.-H.; Hsu, H.-F., Direct C–H Arylation Polymerization to form Anionic Water-Soluble Poly(3,4-ethylenedioxythiophenes) with Higher Yields and Molecular Weights. *Synlett* **2018,** *29,* 2660-2668.
3. Yano, H.; Kudo, K.; Marumo, K.; Okuzaki, H., Fully soluble self-doped poly(3,4-ethylenedioxythiophene) with an electrical conductivity greater than 1000 S cm^{-1}. *Sci. Adv.* **2019,** *5,* eaav9492.
4. Otero, M.; Dittrich, T.; Rappich, J.; Heredia, D. A.; Fungo, F.; Durantini, E.; Otero, L., Photoinduced charge separation in organic-inorganic hybrid system: C60-containing electropolymer / CdSe-quantum dots. *Electrochim. Acta* **2015,** *173,* 316-322.

5. Karlsson, R. H.; Herland, A.; Hamedi, M.; Wigenius, J. A.; Åslund, A.; Liu, X.; Fahlman, M.; Inganäs, O.; Konradsson, P., Iron-Catalyzed Polymerization of Alkoxysulfonate-Functionalized 3,4-Ethylenedioxythiophene Gives Water-Soluble Poly(3,4-ethylenedioxythiophene) of High Conductivity. *Chem. Mater.* **2009,** *21,* 1815-1821.
6. Zotti, G.; Zecchin, S.; Schiavon, G.; Groenendaal, L. B., Electrochemical and chemical synthesis and characterization of sulfonated poly(3,4-ethylenedioxythiophene): A novel water-soluble and highly conductive conjugated oligomer. *Macromol. Chem. Phys.* **2002,** *203,* 1958-1964.
7. Cutler, C. A.; Bouguettaya, M.; Kang, T.-S.; Reynolds, J. R., Alkoxysulfonate-Functionalized PEDOT Polyelectrolyte Multilayer Films: Electrochromic and Hole Transport Materials. *Macromolecules* **2005,** *38,* 3068-3074.
8. Rivnay, J.; Leleux, P.; Sessolo, M.; Khodagholy, D.; Hervé, T.; Fiocchi, M.; Malliaras, G. G., Organic Electrochemical Transistors with Maximum Transconductance at Zero Gate Bias. *Adv. Mater.* **2013,** *25,* 7010-7014.

WinGPC UniChrom Analysis Report

A5 Sample: Sample 1 212333

Method Information

Project:	W:\GPC_DATEN\AN\LC\serv_21\serv21_PG07.LDX	Injection time:	10/18/2021 10:49:48
GPC instrument:	PG07	Operator:	MM
Calibration type:	Conventional	Calibration fit:	Polynomial 3
Calibration file:	PSS_1018_MCX_A.CAL	Int. standard:	System
Vp int. standard calib.:	25.43 mL	Vp int. standard sample:	0.00 mL
Injection volume:	50 µL	Autosampler temp.:	-
Sample concentration:	1.000 g/L	Column temperature:	70.0 °C
Eluent:	0.07 M Na2HPO4 aq	Flow rate:	1.00 mL/min
Columns:	PSS MCX, 10 µm, Guard + 1,000 Å + 100,000 Å		

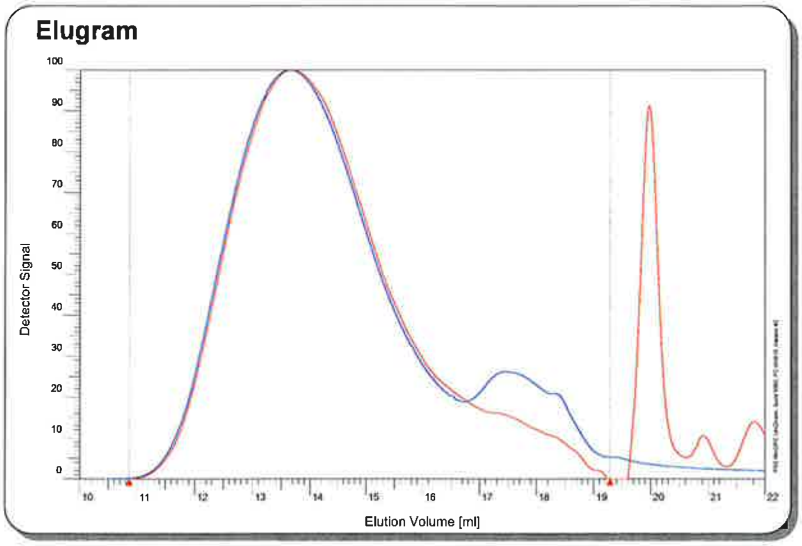

Baseline from: 10.46 mL to: 30.37 mL
Integration from: 10.86 mL to: 19.28 mL

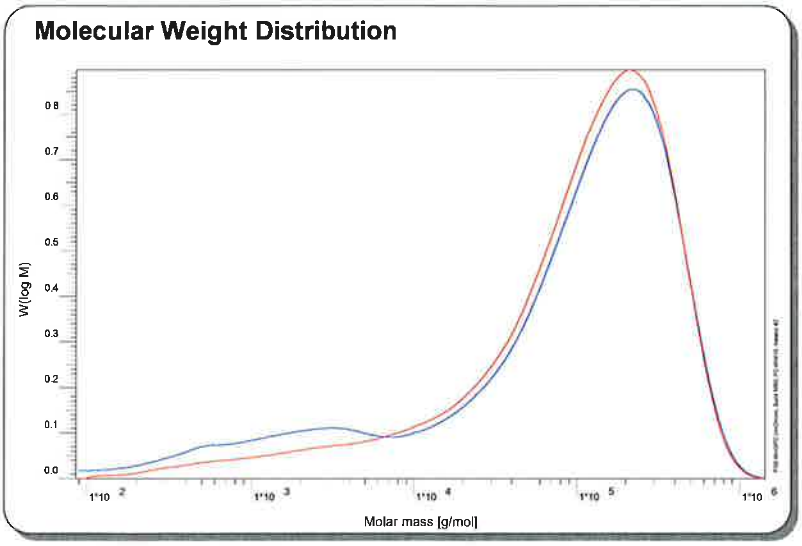

	Detector	Mn /Da	Mw /Da	Mz /Da	PDI (=Mw/Mn)	Vp /mL	Mp /Da	Area
blue	UV@254 nm	5080	174000	344000	34.26	13.65	188000	168.8700
red	RID	10200	178000	333000	17.49	13.67	186000	8.3106

WinGPC UniChrom Analysis Report

Konradsson Sample: Sample 3 212335

PSS

Method Information

Project:	W:\GPC_DATEN\AN\LC\serv_21\serv21_PG07.LDX	Injection time:	10/18/2021 12:25:54
GPC instrument:	PG07	Operator:	MM
Calibration type:	Conventional	Calibration fit:	Polynomial 3
Calibration file:	PSS_1018_MCX_A.CAL	Int. standard:	System
Vp int. standard calib.:	25.43 mL	Vp int. standard sample:	0.00 mL
Injection volume:	50 µL	Autosampler temp.:	-
Sample concentration:	1.000 g/L	Column temperature:	70.0 °C
Eluent:	0.07 M Na2HPO4 aq	Flow rate:	1.00 mL/min
Columns:	PSS MCX, 10 µm, Guard + 1,000 Å + 100,000 Å		

Elugram

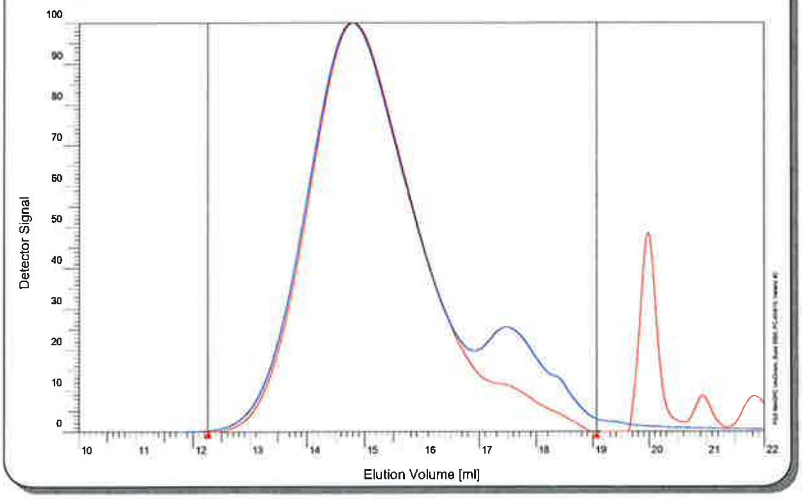

Baseline from: 11.78 mL to: 27.94 mL
Integration from: 12.26 mL to: 19.07 mL

Molecular Weight Distribution

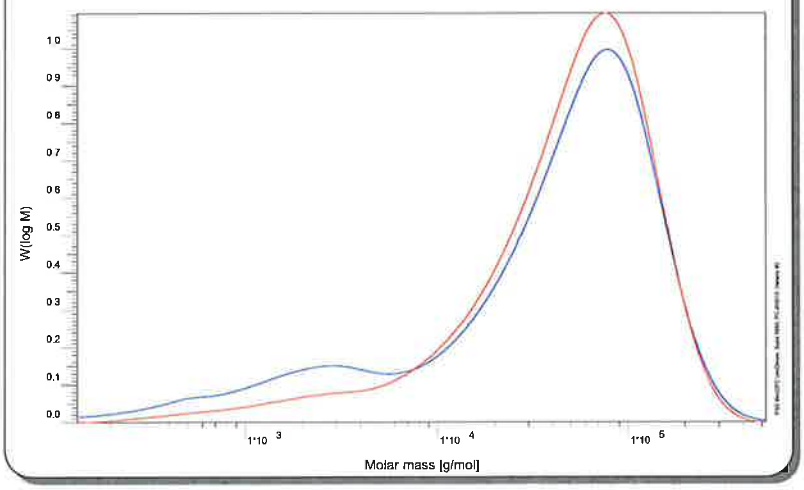

	Detector	Mn /Da	Mw /Da	Mz /Da	PDI (=Mw/Mn)	Vp /mL	Mp /Da	Area
blue line	UV@254 nm	6040	66100	123000	10.95	14.79	70700	267.7770
red line	RID	13000	69300	116000	5.33	14.82	68500	13.5144

WinGPC UniChrom Analysis Report

Sautter Sample: Sample 4 212336

PSS

Method Information

Project:	W:\GPC_DATEN\AN\LC\serv_21\serv21_PG07.LDX	Injection time:	10/18/2021 13:24:11
GPC instrument:	PG07	Operator:	MM
Calibration type:	Conventional	Calibration fit:	Polynomial 3
Calibration file:	PSS_1018_MCX_A.CAL	Int. standard:	System
Vp int. standard calib.:	25.43 mL	Vp int. standard sample:	0.00 mL
Injection volume:	50 µL	Autosampler temp.:	-
Sample concentration:	1.000 g/L	Column temperature:	70.0 °C
Eluent:	0.07 M Na2HPO4 aq	Flow rate:	1.00 mL/min
Columns:	PSS MCX, 10 µm, Guard + 1,000 Å + 100,000 Å		

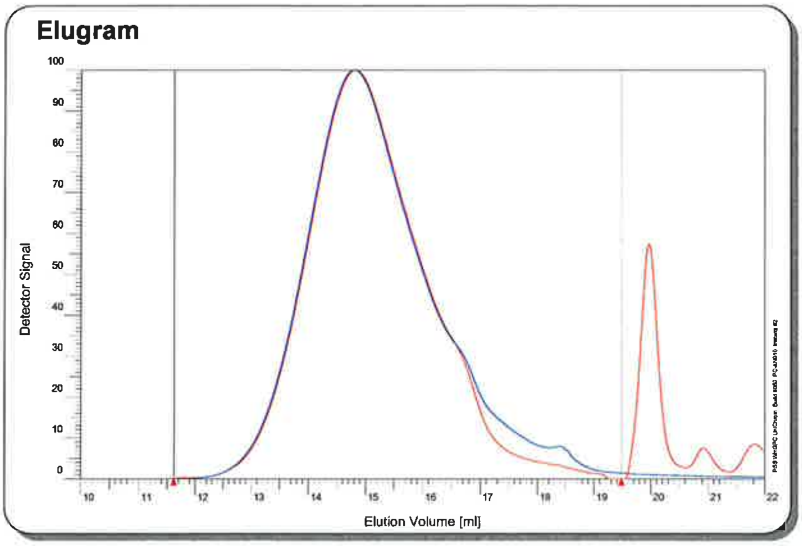

Baseline from: 11.45 mL to: 27.80 mL
Integration from: 11.62 mL to: 19.48 mL

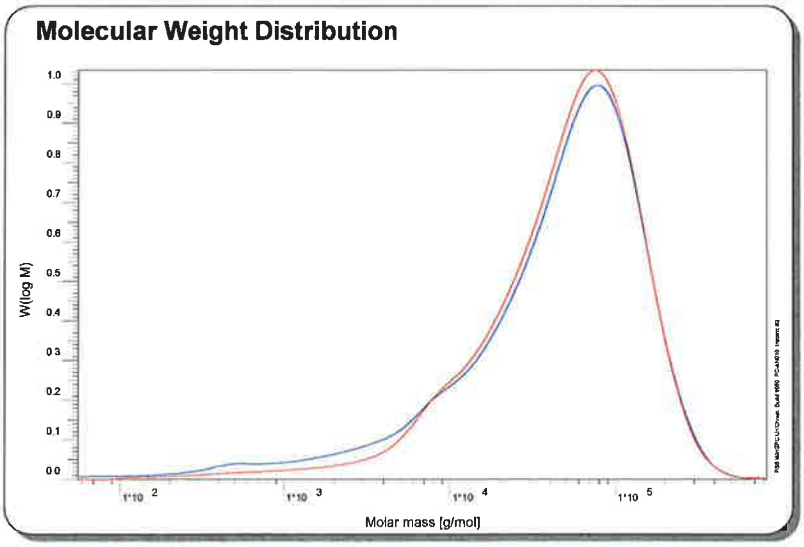

	Detector	Mn /Da	Mw /Da	Mz /Da	PDI (=Mw/Mn)	Vp /mL	Mp /Da	Area
——	UV@254 nm	7190	71700	133000	9.96	14.79	70900	310.8190
——	RID	13100	73300	133000	5.60	14.82	68800	18.0803

WinGPC UniChrom Analysis Report

A5 + 10% EDOT-OH Sample: Sample 5 212337

Method Information

Project:	W:\GPC_DATEN\AN\LC\serv_21\serv21_PG07.LDX	Injection time:	10/18/2021 14:03:44
GPC instrument:	PG07	Operator:	MM
Calibration type:	Conventional	Calibration fit:	Polynomial 3
Calibration file:	PSS_1018_MCX_A.CAL	Int. standard:	System
Vp int. standard calib.:	25.43 mL	Vp int. standard sample:	0.00 mL
Injection volume:	50 µL	Autosampler temp.:	-
Sample concentration:	1.000 g/L	Column temperature:	70.0 °C
Eluent:	0.07 M Na2HPO4 aq	Flow rate:	1.00 mL/min
Columns:	PSS MCX, 10 µm, Guard + 1,000 Å + 100,000 Å		

Elugram

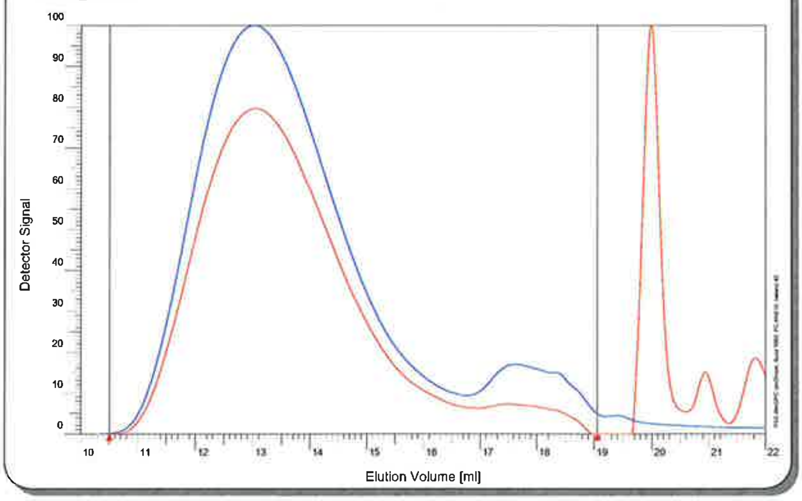

Baseline from: 10.29 mL to: 30.05 mL
Integration from: 10.51 mL to: 19.06 mL

Molecular Weight Distribution

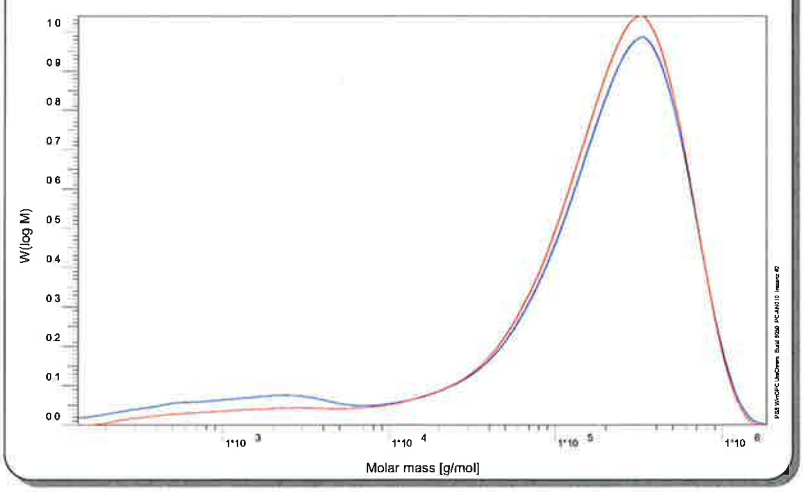

	Detector	Mn /Da	Mw /Da	Mz /Da	PDI (=Mw/Mn)	Vp /mL	Mp /Da	Area
——	UV@254 nm	7630	277000	494000	36.27	13.02	303000	208.6710
——	RID	17100	284000	480000	16.66	13.07	292000	10.8753

WinGPC UniChrom Analysis Report

Sample: Sample 6 212338

A5 without Fe in the synthesis

Method Information

Project:	W:\GPC_DATEN\AN\LC\serv_21\serv21_PG07.LDX	Injection time:	10/18/2021 14:48:27
GPC instrument:	PG07	Operator:	MM
Calibration type:	Conventional	Calibration fit:	Polynomial 3
Calibration file:	PSS_1018_MCX_A.CAL	Int. standard:	System
Vp int. standard calib.:	25.43 mL	Vp int. standard sample:	0.00 mL
Injection volume:	50 µL	Autosampler temp.:	-
Sample concentration:	1.000 g/L	Column temperature:	70.0 °C
Eluent:	0.07 M Na2HPO4 aq	Flow rate:	1.00 mL/min
Columns:	PSS MCX, 10 µm, Guard + 1,000 Å + 100,000 Å		

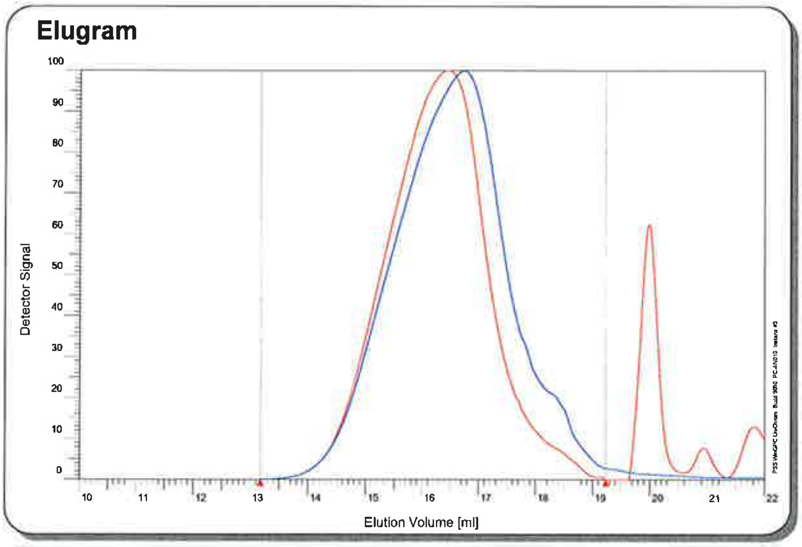

Baseline from: 13.09 mL to: 27.38 mL
Integration from: 13.18 mL to: 19.23 mL

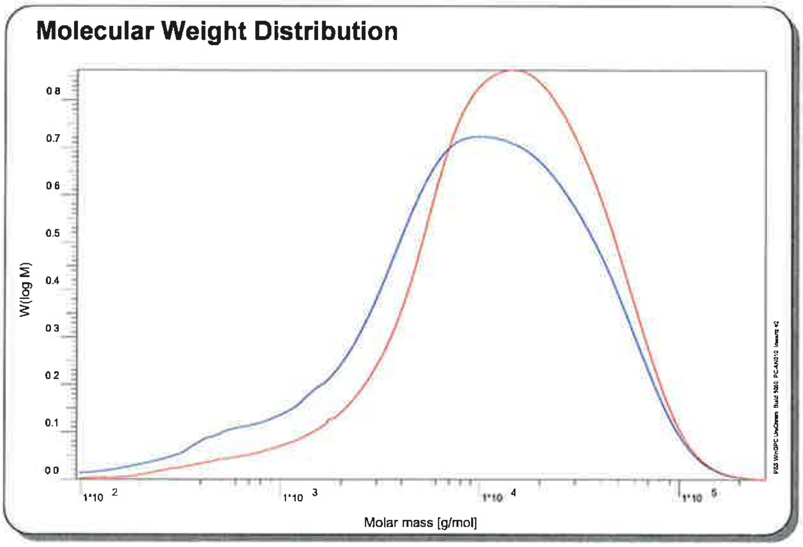

Detector	Mn /Da	Mw /Da	Mz /Da	PDI (=Mw/Mn)	Vp /mL	Mp /Da	Area
UV@254 nm	3240	17900	42900	5.53	16.73	7730	385.8460
RID	5730	21200	43300	3.70	16.47	11000	16.9771

WinGPC UniChrom Analysis Report

Overlay Molecular Weight Distributions

PSS

Method Information

Project:	W:\GPC_DATEN\AN\LC\serv_21\serv21_PG07.LDX	Injection date:	19.10.2021
GPC instrument:	PG07	Operator:	MM
Calibration type:	Conventional	Calibration fit:	Polynomial 3
Calibration file:	PSS_1018_MCX_A.CAL	Int. standard:	System
Vp int. standard calib.:	25,43 mL	Vp int. standard sample:	0,00 mL
Injection volume:	50 µL	Temp. autosampler:	-
Sample concentration:	1,0 g/L	Column temperature:	70,0 °C
Eluent:	0.07 M Na2HPO4 aq	Flow rate:	1,00 mL/min
Columns:	PSS MCX, 10 µm, Guard + 1,000 Å + 100,000 Å		

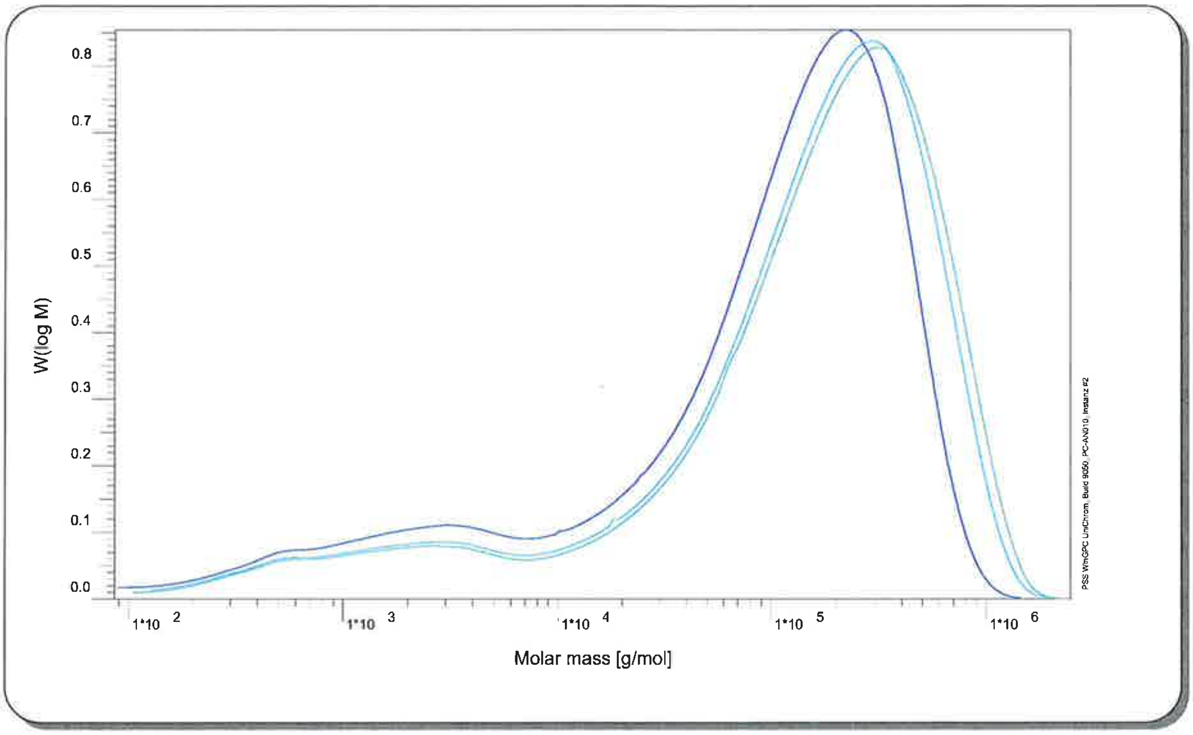

Curve No. 1 : UV@254 nm, Sample 1 212333
Curve No. 2 : UV@254 nm, Sample 1 212333 after 12 h
Curve No. 3 : UV@254 nm, Sample 1 212333 after 24 h

	Curve No.	Mn /Da	Mw /Da	Mz /Da	PDI (=Mw/Mn)	Vp /mL	Mp /Da	Area
——	1	5080	174000	344000	34,26	13,65	188000	168,870000
——	2	7260	245000	487000	33,72	13,30	246000	214,538000
——	3	7420	269000	537000	36,34	13,20	266000	224,023000

WinGPC UniChrom Analysis Report

Overlay Molecular Weight Distributions

Method Information

Project:	W:\GPC_DATEN\AN\LC\serv_21\serv21_PG07.LDX	Injection date:	19.10.2021
GPC instrument:	PG07	Operator:	MM
Calibration type:	Conventional	Calibration fit:	Polynomial 3
Calibration file:	PSS_1018_MCX_A.CAL	Int. standard:	System
Vp int. standard calib.:	25,43 mL	Vp int. standard sample:	0,00 mL
Injection volume:	50 µL	Temp. autosampler:	-
Sample concentration:	1,0 g/L	Column temperature:	70,0 °C
Eluent:	0.07 M Na2HPO4 aq	Flow rate:	1,00 mL/min
Columns:	PSS MCX, 10 µm, Guard + 1,000 Å + 100,000 Å		

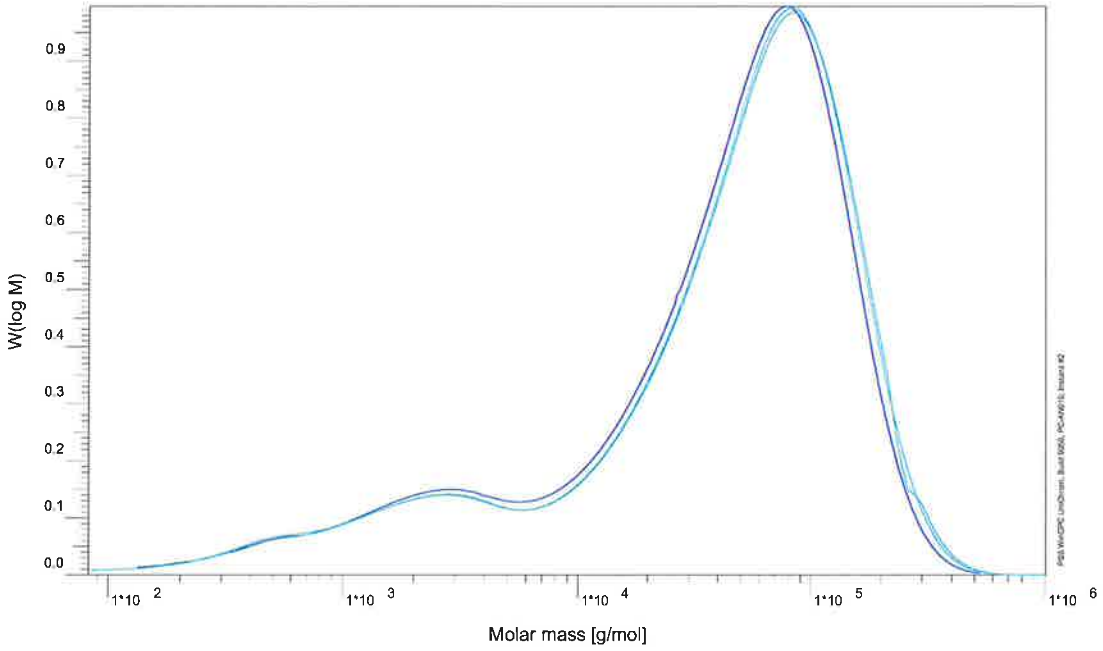

Curve No. 1 : UV@254 nm, Sample 3 212335
Curve No. 2 : UV@254 nm, Sample 3 212335 after 12 h
Curve No. 3 : UV@254 nm, Sample 3 212335 after 24 h

	Curve No.	Mn /Da	Mw /Da	Mz /Da	PDI (=Mw/Mn)	Vp /mL	Mp /Da	Area
——	1	6040	66100	123000	10,95	14,79	70700	267,777000
——	2	6450	71700	134000	11,11	14,73	74500	276,246000
——	3	5480	72300	136000	13,19	14,71	76100	278,466000

WinGPC UniChrom Analysis Report

Overlay Molecular Weight Distributions

Method Information

Project:	W:\GPC_DATEN\AN\LC\serv_21\serv21_PG07.LDX	Injection date:	19.10.2021
GPC instrument:	PG07	Operator:	MM
Calibration type:	Conventional	Calibration fit:	Polynomial 3
Calibration file:	PSS_1018_MCX_A.CAL	Int. standard:	System
Vp int. standard calib.:	25,43 mL	Vp int. standard sample:	0,00 mL
Injection volume:	50 µL	Temp. autosampler:	-
Sample concentration:	1,0 g/L	Column temperature:	70,0 °C
Eluent:	0.07 M Na2HPO4 aq	Flow rate:	1,00 mL/min
Columns:	PSS MCX, 10 µm, Guard + 1,000 Å + 100,000 Å		

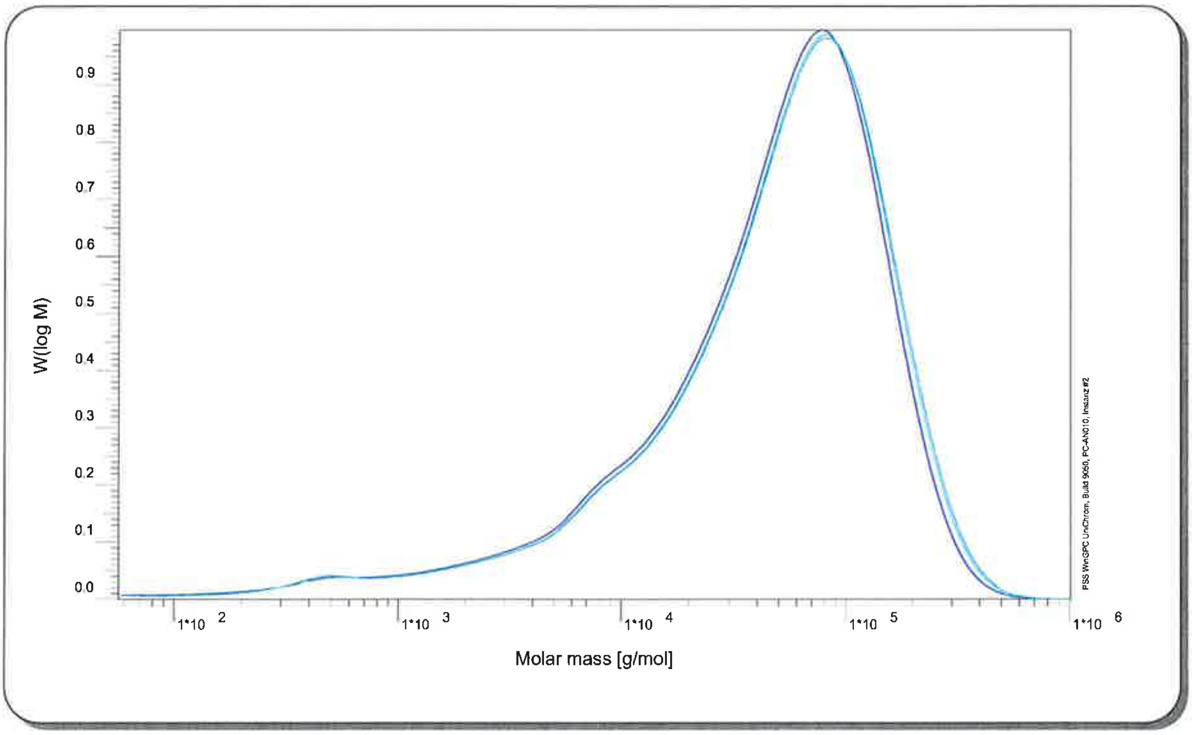

Curve No. 1 : UV@254 nm, Sample 4 212336
Curve No. 2 : UV@254 nm, Sample 4 212336 after 12 h
Curve No. 3 : UV@254 nm, Sample 4 212336 after 24 h

	Curve No.	Mn /Da	Mw /Da	Mz /Da	PDI (=Mw/Mn)	Vp /mL	Mp /Da	Area
——	1	7190	71700	133000	9,96	14,79	70900	310,819000
——	2	8460	76000	142000	8,99	14,77	71900	318,041000
——	3	9870	77400	145000	7,84	14,76	73100	320,035000

WinGPC UniChrom Analysis Report

Overlay Molecular Weight Distributions

Method Information

Project:	W:\GPC_DATEN\AN\LC\serv_21\serv21_PG07.LDX	Injection date:	19.10.2021
GPC instrument:	PG07	Operator:	MM
Calibration type:	Conventional	Calibration fit:	Polynomial 3
Calibration file:	PSS_1018_MCX_A.CAL	Int. standard:	System
Vp int. standard calib.:	25,43 mL	Vp int. standard sample:	0,00 mL
Injection volume:	50 µL	Temp. autosampler:	-
Sample concentration:	1,0 g/L	Column temperature:	70,0 °C
Eluent:	0.07 M Na2HPO4 aq	Flow rate:	1,00 mL/min
Columns:	PSS MCX, 10 µm, Guard + 1,000 Å + 100,000 Å		

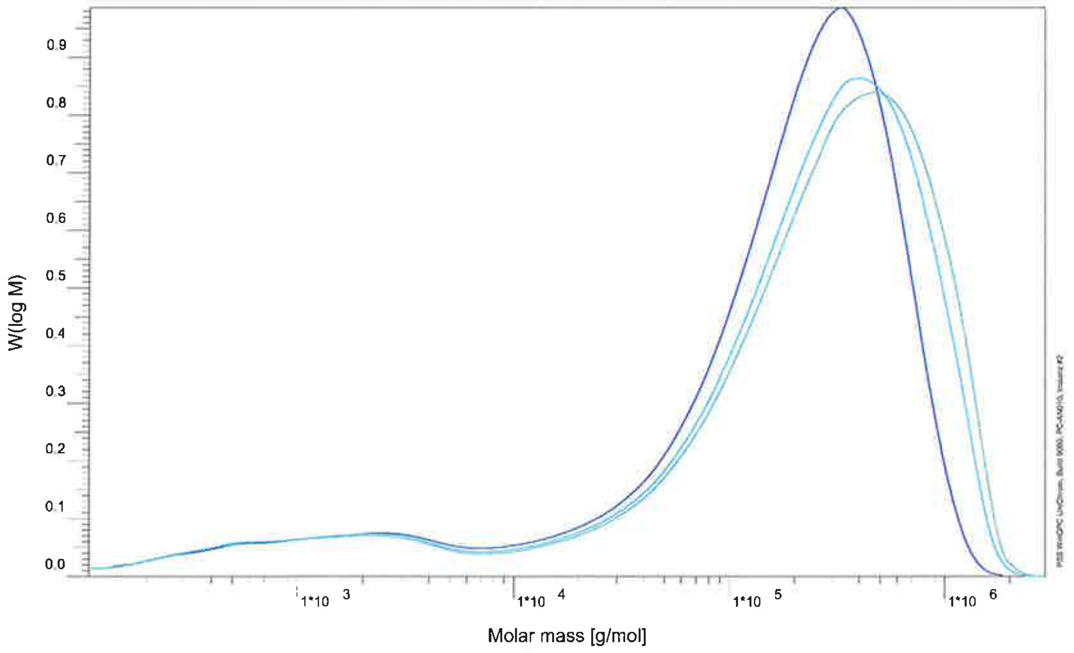

Curve No. 1 : UV@254 nm, Sample 5 212337
Curve No. 2 : UV@254 nm, Sample 5 212337 after 12 h
Curve No. 3 : UV@254 nm, Sample 5 212337 after 24 h

	Curve No.	Mn /Da	Mw /Da	Mz /Da	PDI (=Mw/Mn)	Vp /mL	Mp /Da	Area
——	1	7630	277000	494000	36,27	13,02	303000	208,671000
——	2	6860	360000	676000	52,52	12,64	399000	210,318000
——	3	7320	400000	747000	54,60	12,41	469000	218,195000

WinGPC UniChrom Analysis Report

Overlay Molecular Weight Distributions

Method Information

Project:	W:\GPC_DATEN\AN\LC\serv_21\serv21_PG07.LDX	Injection date:	19.10.2021
GPC instrument:	PG07	Operator:	MM
Calibration type:	Conventional	Calibration fit:	Polynomial 3
Calibration file:	PSS_1018_MCX_A.CAL	Int. standard:	System
Vp int. standard calib.:	25,43 mL	Vp int. standard sample:	0,00 mL
Injection volume:	50 µL	Temp. autosampler:	-
Sample concentration:	1,0 g/L	Column temperature:	70,0 °C
Eluent:	0.07 M Na2HPO4 aq	Flow rate:	1,00 mL/min
Columns:	PSS MCX, 10 µm, Guard + 1,000 Å + 100,000 Å		

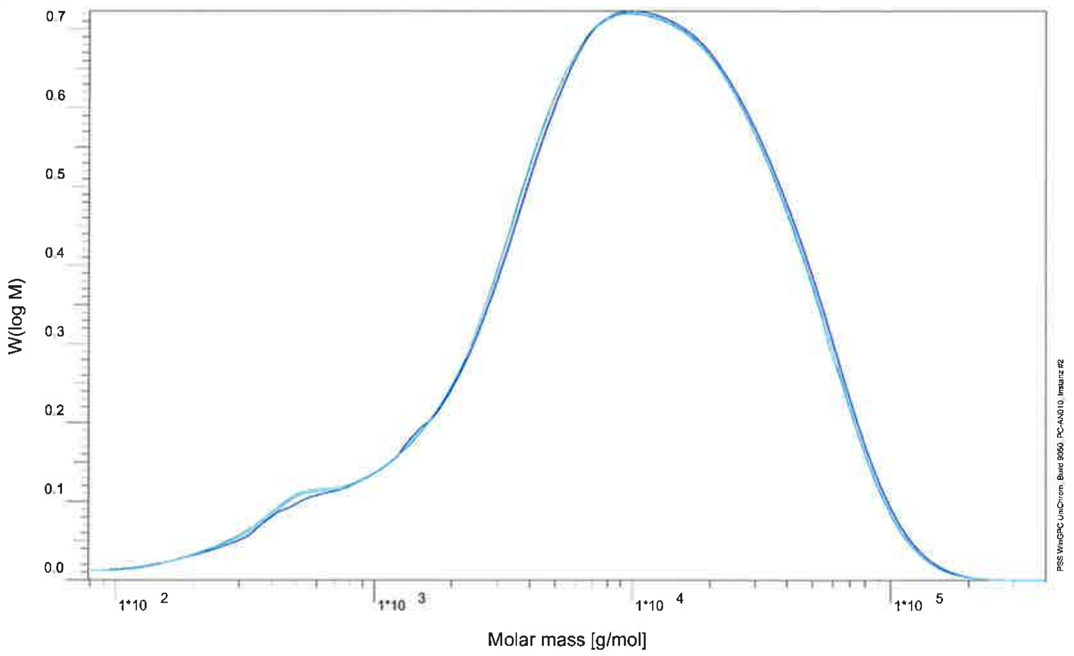

Curve No. 1 : UV@254 nm, Sample 6 212338
Curve No. 2 : UV@254 nm, Sample 6 212338 after 12 h
Curve No. 3 : UV@254 nm, Sample 6 212338 after 24 h

	Curve No.	Mn /Da	Mw /Da	Mz /Da	PDI (=Mw/Mn)	Vp /mL	Mp /Da	Area
	1	3240	17900	42900	5,53	16,73	7730	385,846000
	2	3170	17500	42000	5,52	16,75	7560	389,892000
	3	3030	17300	41600	5,72	16,77	7370	392,000000
